# Creating complete life histories of individual female tsetse (*Glossina* spp) to study the effects of meteorological conditions on fly size in Zimbabwe

**DOI:** 10.1371/journal.pntd.0014120

**Published:** 2026-08-19

**Authors:** John W. Hargrove, Faikah Bruce, John Van Sickle

**Affiliations:** 1 South African Centre for Epidemiological Modelling and Analysis (SACEMA), Centre for Epidemic Response and Innovation (CERI), School for Data Science and Computational Thinking, Stellenbosch University, Stellenbosch, South Africa; 2 (Retired) Department of Fisheries, Wildlife and Conservation Sciences, Oregon State University, Corvallis, Oregon, United States of America; University of Pretoria, SOUTH AFRICA

## Abstract

Using novel methodologies and ovarian dissection, we constructed life histories for ~90,000 female *Glossina pallidipes* and *G. m. morsitans* collected in Zimbabwe (1988–1999). Temperature-dependent development rates were used to estimate, for each fly, the dates spent as an oocyte, egg, larva, pupa – and adult in each ovarian category. This enabled analysis of relationships between wing and egg lengths of field-captured adults – and mean environmental conditions prevailing when these lengths were being determined, during oogenesis for eggs, and oogenesis plus pregnancy for wings. Lengths all oscillated seasonally with well-marked minima in the hot-dry season, when selection against small individuals continued for some weeks after emergence. Lengths were negatively associated with temperature and positively with Normalized Difference Vegetation Index (NDVI), which measures plant health, density, and vigor. Multiple linear regression models had R^2^ values of 0.68 and 0.66 for the means of egg and wing lengths, respectively, of flies pooled weekly in *G. pallidipes*. Corresponding R^2^ values were 0.53 and 0.56 for monthly pooling in *G. m. morsitans*. Mean wing length in *G. pallidipes* also increased with increasing rainfall. Mean egg lengths increased with maternal wing length, were shorter in primiparous flies but changed little thereafter. Factors reflecting moisture, heat and vegetative growth influence tsetse size, survival and abundance indirectly, through effects on vertebrate host availability and feeding risk. Models developed using flies captured after November 1991 accurately predict, with no further calibration, mean egg and wing lengths of flies captured before November 1991. The models facilitate true predictions of future changes in mean fly size based on readily available meteorological data, benefiting vector and disease control efforts in predicting climate-related changes in the vigor of tsetse populations. This aids proactive identification of priority areas for monitoring and control efforts, improving cost-efficacy of tsetse and trypanosomiasis control.

## Author summary

The physical size of a female tsetse is constant during adult life and reflects the conditions experienced while she was developing in her mother’s ovaries and uterus. For all but the youngest adult flies sampled in the field it is not easy to decide when said fly was *in utero*. Using ovarian dissection we can, however, decide how many times the fly had ovulated. We then use established rates of development of different life stages to “wind back the developmental clock” through various pregnancies, to the time when the fly emerged from the puparial case, then back further to the larviposition date, and the dates of the end and start of maternal pregnancy and oogenesis. We find fly size is positively correlated with measures of plant growth, and negatively with temperature, during the time the fly was in its mother’s abdomen. We suggest an indirect causal chain whereby increasing temperatures mean tsetse must feed more frequently, but decreasing food availability for mammals reduces local host density. These factors mean flies suffer increased mortality associated with feeding and produce smaller pupae. We believe that this is the first time that such life histories have been constructed for any insect species.

## Introduction

Adult tsetse flies (*Glossina* spp, Diptera: Glossinidae) are the vectors of human and animal trypanosomiasis (HAT and AAT) in Africa, and their field biology has been the subject of intense study since the early 20^th^ century. There have been impressive strides recently in the elimination of the Gambian form of trypanosomiasis and vector control plays an important part in this process [1–3]. Improved understanding of tsetse biology and population dynamics can thus continue to play a significant role in disease control.

Reproduction in tsetse involves the production, at approximately 9-day intervals, of a single larva that weighs as much or more than its postpartum mother [4–6]. Once deposited the larva does not feed. Instead, it burrows into loose soil then forms around itself a hard puparial case, within which metamorphosis is completed. The teneral adult fly that emerges from the puparial case, and hardens, has the linear dimensions of the mature adult. The size of the wing, which is commonly used as a measure of fly size, is fully determined by the start of the pupal stage. Thereafter, wing size does not increase with age and only shortens in the adult stage due to wearing at the wing margins. Similarly, the length of an egg is fixed by the time it is ovulated into the uterus [7].

As with all insects, the size of individuals of a given species of tsetse (*Glossina* spp) is constrained within relatively tight limits though individual sizes vary consistently with season. It has been argued that size variation reflects the physical conditions experienced by the individual’s mother while she was bearing the oocytes, egg and larval stages [8–16]. The central problem that bedevils all such studies was recognised by Glasgow and Bursell [11] who noted that “The demonstration of a significant correlation should be the beginning, not the end, of such investigations, since it is essential to establish whether the causal chain postulated as linking the variables does in fact exist”; 64 years later no such causal link has yet been established.

Jackson [9] used wing fray measurements to estimate a mean age of 3–4 weeks for male tsetse collected near Shinyanga in Tanzania [17,18]. Since the pupal period is about 35 days for males at the prevailing mean temperature of 23°C, he expected that the size of male flies of average age 3–4 weeks would be determined approximately 2 months prior to collection, at the time when the fly’s mother was pregnant. He found a negative correlation between mean wing vein length and mean saturation deficit 2 months prior to capture. Saturation deficit is the difference between the amount of moisture air can hold when saturated and the amount present in the air at a given temperature. It provides an index of the air’s drying power. This correlation was strongest for *G. pallidipes* Austen but was also significant for *G. swynnertoni* Austen and *G. m. morsitans* Westwood. Correlations were progressively reduced as the time lag was reduced to one month and zero months and the correlation was weaker with temperature than with saturation deficit. Note that, whereas all males were assigned the average age of 3–4 weeks, some flies will have been *in utero* for less and some for more time, than the two-month lag period assumed for all flies.

The studies mentioned above all involved the analysis of male tsetse, since females were much more difficult to sample using the handnet method of sampling used at the time. Females are of greater interest, however, and with the advent of odor-baited traps were readily captured. Dransfield and co-workers [15] analyzed newly emerged female *G. pallidipes* trapped at Nguruman, Kenya, close to the equator. Assuming constant durations of pregnancy and pupal duration throughout the year, they estimated that pregnancy occurred approximately two months prior to sampling. Wing lengths were positively correlated with minimum relative humidity and negatively correlated with maximum temperature, maximum saturation deficit and mean soil temperature. The advantage of this study was that it pinpointed the timing of pregnancy by using flies of known age; its shortcoming was that it used information only from the youngest female flies, which generally comprise <7% of flies sampled using traps [19].

In this paper, we take up the challenge of relating fly sizes to environmental factors. We introduce new methodologies that allow us to estimate entire life histories, for each of 87,000 field-captured female tsetse, and thereby identify the period during which each of a very large number of individuals was developing in its mother’s ovaries and uterus. We then compute averages of meteorological variables over this developmental period for each fly and use correlation and regression to relate these averages to fly size. We included all females for which the chronological age and pupal duration could be estimated and have developed a method for estimating the calendar period when that fly was developing inside its mother, either as an oocyte in the ovaries, or as an egg or larva *in utero*. This has been achieved using subsets of data arising from an 11-year study involving the ovarian dissection of *ca*. 154, 500 *G. pallidipes* and *ca*. 19, 500 *G. m. morsitans*. Since the work was performed in Zimbabwe, relatively far from the equator, seasonal variation in meteorological conditions was much greater than in the East African localities of the studies cited above.

## Methods

### Meteorological measurements

Daily maximum and minimum temperatures were recorded from a mercury thermometer in a Stevenson screen at Rekomitjie; a rain gauge sited next to the screen, likewise, produced daily records of precipitation. From 17 November 1991 – 31 December 1999, hourly mean measurements of shade temperature and relative humidity were also made using an automatic weather station (type WS01, Delta-T devices, Newmarket, UK) sited *ca*. 200 m from the Stevenson screen. Temperature and relative humidity data from this logger were used to calculate the saturation deficit – an index of humidity characterized by the difference between the saturation vapor pressure and the actual vapor pressure of a volume of air. Saturation deficit is thus a pressure, with units conveniently expressed in millibars (mB) and has the property of being proportional to the evaporation capability of the air.

We also estimated the number of hours available for tsetse feeding each day, as a potential explanatory variable. This variable may alter the probability that a female fly finds and feeds on a host on a given day – and may therefore impact her nutrition during pregnancy. Tsetse are generally inactive at night, and at low and high temperatures [20]. Accordingly, any hour of a given day was judged suitable for tsetse feeding activity if it lay between the recorded times of sunrise and sunset at Rekomitjie and if the mean temperature for that hour was ≥ 16 and ≤ 32°C.

Daily means of the hourly readings from the logger were calculated for temperature, relative humidity and saturation deficit, and were denoted as *Tbar*, *RHbar* and *SDbar*, respectively. Monthly values for the Normalized Difference Vegetation Index (NDVI), were obtained from data published by the *National Oceanic and Atmospheric Administration* (NOAA), or from Modis if NOAA figures were not available. Monthly measures were made for an area within 2km of the Research Station offices. For modelling purposes, monthly NDVI figures were used to generate daily estimates, using linear interpolation.

Data from the logger were the most useful for our purpose since, in addition to the temperature and rainfall data available from the Stevenson screen, the logger also produced measures of relative humidity and, thereby, saturation deficit. Accordingly, some of our statistical models used biological data produced between 17 November 1991 and 31 December 1999, when several meteorological variables were available from the logger, to serve as candidate explanatory variables. We could not, however, apply such models outside this period, before logger data were available. Accordingly, we also developed predictive models that used only NDVI and data available from the Stevenson screen, all of which were available during the whole study period (S1 Data).

### Capture and processing of female tsetse

All field studies were carried out at Rekomitjie Research Station (16°10′S, 29° 25′E, altitude 520 m), Zambezi Valley, Zimbabwe. Methods associated with the capture, ovarian dissection and measurement of wing length and wing fray of female tsetse have been detailed in numerous published papers [4,5,16,19,21–32]. Accordingly, the following provides only a summary of methods described elsewhere, but with fuller descriptions of the methods developed specifically for the present analyses. Note that, in this paper, we are only concerned with analyses of female tsetse – particularly since it is not currently possible to provide accurate estimates of the age of male flies.

### Ovarian dissection

Between September 1988 and December 1999, field-caught tsetse, collected in cages or in glass or Perspex tubes (75 x 25 mm), were placed under a moist black cloth in a polystyrene box and transferred to a field laboratory for further processing. Adult females were subjected to ovarian dissection (*ca*. 98% within 24 hours of capture) and assigned to one of eight ovarian categories, using the disposition and relative sizes of the oocytes within the ovarioles in the left and right ovaries. This procedure can be used to determine unequivocally the number of times a fly has ovulated if, and only if, she is in her first ovarian cycle – *i.e*., has ovulated fewer than four times [33] (Fig A in S1 Appendix). For older flies, we cannot differentiate, using only ovarian dissection data, between flies in their second and later ovarian cycles. Thus, we cannot distinguish flies that have ovulated 4 times from those that have ovulated 8, 12, 16 *etc*. times – and similar problems exist for those that have ovulated 5, 6 or 7 times.

We are specifically interested in the factors affecting the physical size of a fly, as judged by the lengths of the *in utero* egg, and/or of the wing, as measured using a binocular microscope. Egg length was measured after the egg was dissected out of the uterus. Wing length was taken as the distance between landmarks 1 and 6 as defined in [34]. This distance is less than the total length of a wing in perfect condition. It is chosen, however, because wing tips are frequently damaged, making it impossible to obtain consistent, accurate measures of the full length.

#### Temperature dependent durations of oogenesis, pregnancy and pupal development.

Development rates in tsetse are strongly temperature dependent at all stages of life. Table 1 provides estimates, for female *G. m. morsitans* and *G. pallidipes*, of the temperature-dependent daily rates of passage to first ovulation, through subsequent pregnancies and during all development that occurs within the puparial case. Rates of *G. m. morsitans* pupal development were studied in detail by [37]. No such data are available for other species of tsetse species, and we assume identical pupal development rates for *G. pallidipes*. We also assume that, as a mature oocyte is ovulated into the uterus, a new oocyte starts developing and is itself ready to be ovulated shortly after the next larviposition. For each of these developmental stages, we estimated the proportion of development completed on a given day from the mean temperature for that day, based on Table 1. The proportions completed were then accumulated over consecutive days until the first day when the cumulative proportions exceeded a value of 1.0, thereby defining the estimated number of days required to complete the developmental stage in question.

**Table 1:** Estimated rates of development of different life stages of female tsetse. All rates have units of the proportion of the life stage completed, per day. Temperatures (*Tbar*) in the Phelps & Burrows [35] study were maintained at constant levels in the laboratory. For the field estimates in this table *Tbar* was taken as the mean of the daily maximum and minimum temperatures (degrees C), measured in a Stevenson screen. The rate *r*_2_ was taken as the inverse of the observed pregnancy duration, which decreased with temperature.

Rate (*r*_0_) Female pupal development	*r*_0_ = *k*_0_/(1+exp(*a*_0_ + *b*_0_*Tbar*))	[36]
	*k*_0_ = 0.05689; *a*_0_ = 5.470; *b*_0_ = -0.2457	[36]
Rate (*r*_1_) passage to first ovulation	*r*_1_ = 0.1428	[25]
Rate (*r*_*2*_) pregnancy progression	*r*_2_ = *a*_2_*+ b*_2_*(*Tbar*-*T*_2_)	[23,24]
	*a*_2_ = 0.1046; *b*_2_ = 0.0052; *T*_2_ = 24	

### Constructing a life history for individual female tsetse

We aimed to estimate meteorological conditions prevailing when individual flies were at various well-defined stages of development, particularly during oogenesis and pregnancy when these conditions should maximally impact the wing length of the developing immature fly, via effects on their mothers. This aim is achieved, using ovarian dissection and meteorological data, as described below. If we know how many times a fly has ovulated, we can estimate its age to the nearest 9–12 days, or to the nearest 3–4 days if we also use the lengths of any larva *in utero* and of the two largest oocytes in the ovaries. This information allows computation of the proportion of pregnancy completed and, given knowledge of the inter-larval period, the estimated number of days since the last ovulation [23,24]. For a fly of known chronological age, and date of capture and dissection, we used temperature profiles over the period of the fly’s life, to reconstruct its life history – using the temperature-dependent rates of development (Table 1) during various phases of the tsetse life cycle. This procedure is best illustrated using a specific example.

### Winding back the clock

Consider a female *G. pallidipes*, captured on 17 July 1993 and found on examination to be in ovarian category *c* = 3. The lengths of the fly’s uterine and ovarian contents were used to estimate that 0.58 of the current pregnancy had been completed – *i.e*., the fly had completed 3.58 ovulations. On 17 July 1993 the mean temperature was 20.9°C and the equation for *r*_2_ in Table 1 predicts that 0.089 of an ovulation cycle was completed on that day, so the fly was estimated to have completed 3.580 - 0.089 = 3.491 ovulations at the start of the previous day, 16 July 1993. The *r*_2_ rate equation, and daily mean temperatures, were used to estimate the proportion of pregnancy completed on each preceding day until the predicted ovulations completed first takes a value ≤ 1.0, which was then taken as the day after the female ovulated for the first time. This event occurred between 18 June, taken as the first day of *c* = 1, and 17 June, taken as the day at the end of which the female ovulated for the first time. The rate *r*_1_ in Table 1 was then used in like manner to estimate that the first day of adult life occurred on 11 June. The adult female thus emerged from its puparial case on 10 June (Table 2) – at the end of which day the pupa was taken to have completed its pupal life. To find the day on which pupal life started, *i.e*., the date of larviposition, we continued the rewinding procedure, using now the temperature-dependent rate *r*_0_ of female pupal development (Table 1). This resulted in a prediction that pupal development started on 5 May, so that 4 May marked the last day of pregnancy, with larviposition estimated to have occurred at the end of that day (Table 2). Finally, we repeated the rewinding procedure, again using the rate *r*_2_, to estimate that the pregnancy producing our example fly spanned the dates 25 April – 4 May, and that the period of oogenesis was 15 – 24 April (Table 2). See Figs B, C in S1 Appendix, for all details of above calculations.

**Table 2:** Mean daily temperatures during different stages of the development of a female *G. pallidipes* captured on 17 July 1993, when she had ovulated three times and had completed an estimated 58% of the current pregnancy. The rows labelled *c* = 0, 1, 2, 3 denote the periods spent between successive ovulations.

Period	Developmental periods	Mean temperature
*c* = 3	11-Jul - 17-Jul	21.79
*c* = 2	30-Jun - 10-Jul	20.15
*c* = 1	18-Jun - 29-Jun	20.25
*c* = 0	11-Jun - 17-Jun	20.76
Pupa	05-May - 10-Jun	23.55
Pregnancy	25-Apr - 04-May	24.46
Oogenesis	15-Apr - 24-Apr	24.80

The same procedure was carried out for all females in their first ovarian cycle – *i.e*., those that had ovulated 0, 1, 2 or 3 times. For flies in their second and later ovarian cycles, ovarian dissection alone cannot be used to say whether a fly is in its second, third or later ovarian cycle. Such flies can only be placed in *c* = 4 + 4*n*, 5 + 4*n*, 6 + 4*n* or 7 + 4*n* where *n* can, in principle, take any non-negative integer value [33]. Wing fray could be used to distribute these older flies between the second, and later, ovarian cycles flies. Applying the procedure to all such flies clearly leads, however, to errors of allocation [27]. Accordingly, we only produced full life histories for flies in ovarian categories *c* = 0–3 – where the problem of age uncertainty does not arise (S2 Data).

### Wing and egg lengths

#### Temporal pooling of wing lengths.

**Fig 1:**
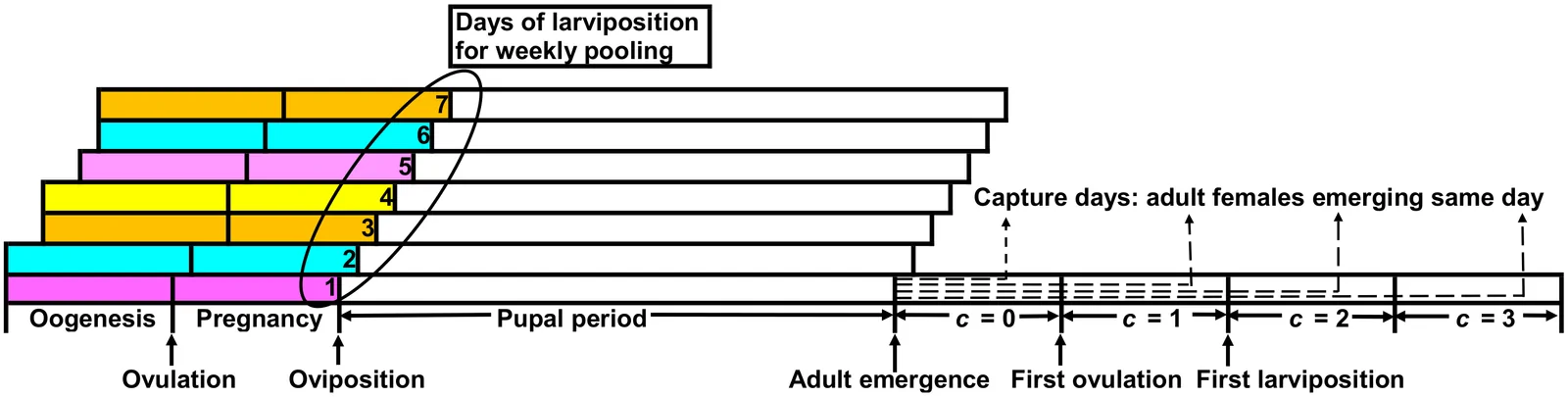
Diagrammatic representation of the procedure for pooling female tsetse, based on their estimated life histories. Examples are given for four sampled flies, found on dissection to be in ovarian categories *c* = 0, 1, 2 and 3, respectively – all of which are estimated to have emerged as adults on the same day. All flies within the black oval can be pooled into one weekly group, based on the end dates of their developmental period: analogous procedures generated data pooled by month.

The procedure described in the previous section identifies daily cohorts of female tsetse, which were deposited as pupae on the same day. Fig 1 illustrates this procedure for flies of different ages, denoted by arrows and dashed lines of different lengths, all of which emerged on day 1 of a week. By assumption, these flies experienced identical meteorological conditions throughout their development – *i.e*., during oogenesis and their mother’s pregnancy. Ideally, one could now relate the mean wing sizes of daily cohorts to summary statistics of meteorological and other environmental variables during their developmental periods. Time series of daily mean wing size were, however, quite noisy due to the large size variability among individual flies, and to small, sometimes zero, catches related to individual days of larviposition. Accordingly, we also pooled flies, based on their larviposition dates, into both monthly and weekly groups. We used monthly pooling to graphically explore coarse annual cycles of fly size and environmental change. Weekly pooling captured rapid changes occurring during some parts of annual cycles. We also used weekly averages from these groups, in our statistical modelling of *G. pallidipes* size versus environmental variables. For *G. m. morsitans* modelling, we used averages from monthly groups, due to our much smaller sample size for this species.

Our weekly pooling approach, illustrated in Fig 1, is based on the life histories of all flies that larviposited over a 7-day period (Monday through the following Sunday) denoted by the numbers in the black oval in Fig 1. All larvae deposited on a given day are assumed to experience identical meteorological conditions during the pupal period and to emerge as adults on the same day. The positions of the arrows indicate the ages at capture of the four example flies, estimated to have been larviposited on day 1 of the week. An analogous situation applies to each day of the week. Weekly means of wing length were calculated as unweighted means, across all flies larviposited during the week.

### Environmental averaging

Our wing size analyses assume that a fly’s eventual size is entirely determined by environmental conditions during the oogenesis and maternal pregnancy (Fig 1). Accordingly, for each fly, we calculated the unweighted mean of daily values, separately for each available meteorological variable, over that fly’s total time interval for those two stages (Table 2, Fig 1). These averaged variables were then used in exploratory correlations and regressions between individual fly sizes and their associated environmental regimes. For weekly and monthly groups of flies, we further averaged the per-fly means of environmental variables, across all flies within each group. This provided weekly and monthly average values of environmental variables, which we then related to corresponding group averages of fly size.

### Egg lengths

We assume that the length of an egg in a female’s uterus depends only on conditions she experienced during the oogenesis period resulting in the production of that egg – *i.e*., the ovulation cycle most recently completed prior to the capture date. We do not, therefore, need a fly’s full life history to determine the oogenesis period of the egg it carries *in utero*. To see this refer to Fig 1, and the three example flies captured where *c* = 1, 2 and 3, respectively. For the fly captured in *c* = 3, we only need to generate the start and end dates for *c* = 2 and analogous statements apply to the flies captured in *c* = 2 and *c* = 1. We could thus also accurately determine development periods for eggs carried by females where *c* = 4 + 4*n* to 7 + 4*n*, even without a reliable estimate of those females’ chronological ages. Accordingly, we included eggs from females of all ovarian categories in our analyses of egg lengths.

We also pooled eggs by week or month, in similar fashion to the pooling done for wing size analyses. Egg-bearing females, and their eggs, were pooled into groups whose end dates of their eggs’ oogenesis stage all fell within the same week (or month). Environmental variables were averaged over the oogenesis period for each egg, and these averages were again averaged over each pooled subset of eggs, in the same fashion as for wing size analyses. Maternal wing length was also averaged across pooled subsets, as another candidate explanatory variable.

### Sample sizes

We used subsets of data from a study carried out between September 1988 and December 1999, during which period 151,269 female *G. pallidipes* and 19,569 female *G. m. morsitans* were subjected to ovarian dissection and provided both a valid ovarian category and a wing length (Table 3, row 1). Throughout the study, daily maximum and minimum temperatures, and rainfall were available from the Stevenson screen and monthly measures of NDVI were accessed from the internet. Relative humidity and saturation deficit were available only from 17 November 1991, for 64% of the dissected *G. pallidipes* and 48% of the *G. m. morsitans* (Row 3). About 51% of all *G. pallidipes*, and 54% of all *G. m. morsitans*, had ovulated fewer than four times and could therefore provide full life histories. We estimated chronological age of each such fly and the period it had spent within its mother’s abdomen during oogenesis and pregnancy (Row 4). Among these flies, about half of each species spent the developmental period at times when meteorological data, including relative humidity, were available from the logger (Row 6). For the analysis of egg lengths (Rows 7–9) it was only necessary that the captured adult female had an egg *in utero*. We did not need her full life history, since we were only interested in the conditions she experienced while the oocyte that gave rise to the egg was developing in her ovary.

**Table 3:** Numbers of adult female tsetse available for analysis of factors affecting wing and egg lengths in *G. pallidipes* and *G. m. morsitans* sampled at Rekomitjie. Flies had a full life history only if they were in ovarian categories *c* = 0 to 3 and if the wing length was recorded. SS indicates that meteorological data were available only from the Stevenson screen; SS/L that they were also available from the logger. *n*/*d* indicates the rows used for numerator and denominator, respectively, when calculating percentages.

Row	Wing length	n/d	*G. pallidipes*	*G. m. morsitans*
1	Total sample		151,269	19,569
2	13-Sep-88–16-Nov-91 SS	2/1	53,954 (36%)	10,083 (52%)
3	17-Nov-91–17-Dec-99 SS/L	3/1	97,315 (64%)	9486 (48%)
4	Full life history available (*c* < 4)	4/1	76,871 (51%)	10,616 (54%)
5	13-Sep-88–16-Nov-91 SS	5/4	28,076 (37%)	6096 (57%)
6	17-Nov-91–17-Dec-99 SS/L	6/4	48,795 (63%)	4520 (43%)
	**Egg length**			
7	Total sample	7/1	65,790 (44%)	6079 (32%)
8	13-Sep-88–16-Nov-91 SS	8/7	25,041 (38%)	3369 (55%)
9	17-Nov-91–17-Dec-99 SS/L	9/7	40,749 (62%)	2710 (45%)

### Statistical methods

#### Relating wing size to environmental factors.

We used linear correlation and multiple linear regression to model wing and egg sizes, using averaged environmental variables as potential explanatory variables. We developed our final predictive models for pooled groups, by using the Akaike Information Criterion (AIC) to help select best-fitting, most parsimonious models [38].

## Results

### Meteorological patterns at Rekomitjie during the study period

**Fig 2:**
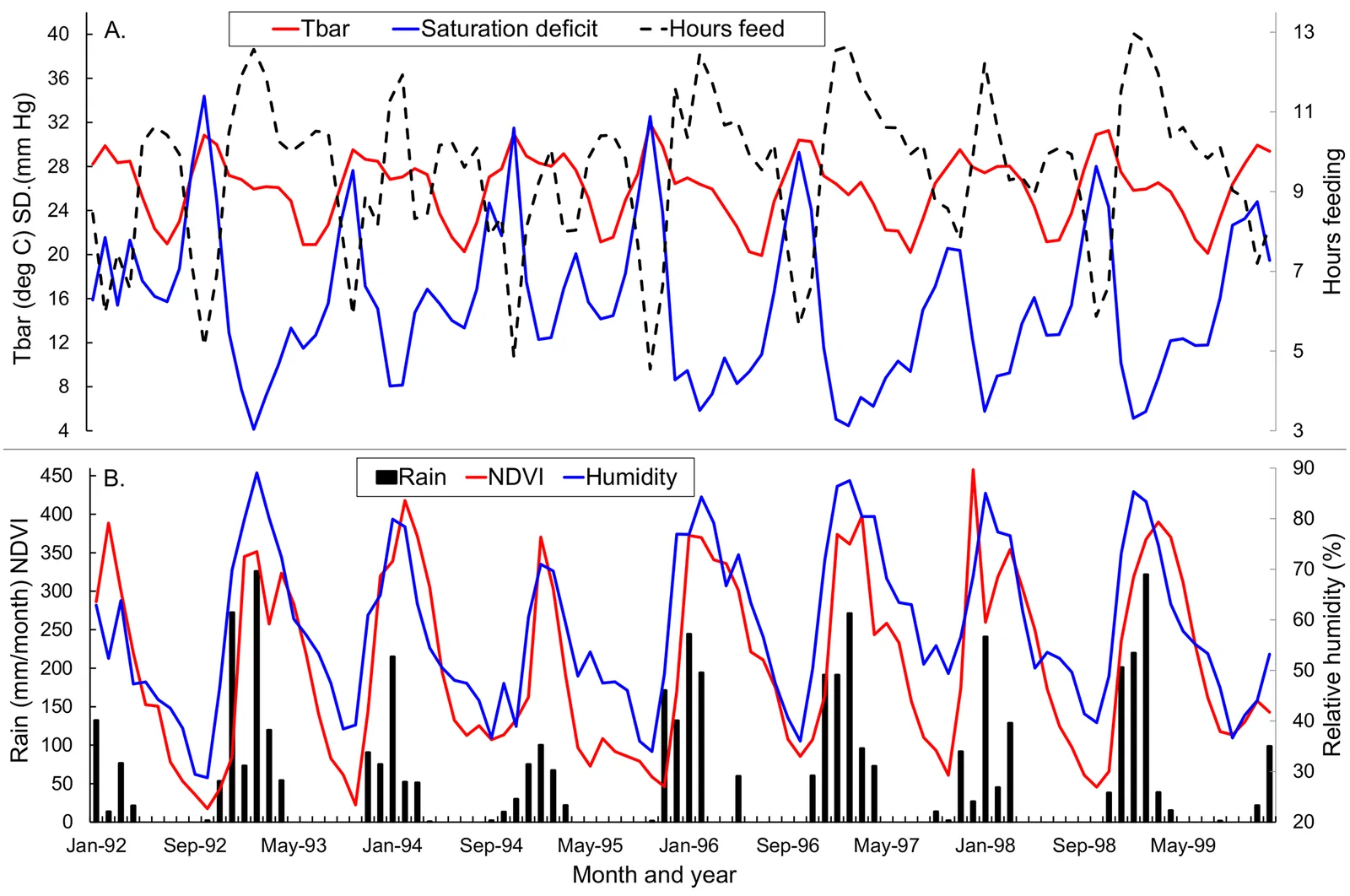
Seasonal changes in meteorological profile 1992-1999. A. Monthly variation in daily mean temperature (*Tbar*; °C), saturation deficit (mB) and the estimated number of hours per day suitable for tsetse feeding activity. B. Total rainfall per month and mean monthly values for the Normalized Difference Vegetation Index (NDVI) and relative humidity over the same period. Values for NDVI, which varied between 0.175 and 0.804, have been scaled for purposes of display.

The general pattern of meteorological changes at Rekomitjie is shown in Fig 2A – and in detail for July 1993-June 1994 in Fig D in S1 Appendix. Temperature and saturation deficit increased steadily, and relative humidity decreased, from July until the rains started in late November/December. Temperatures then fell by 5–10°C but remained at fairly high levels until the end of the rains in March. Temperature and humidity then declined until July, with saturation deficit increasing proportionately. NDVI, which reflects photosynthetic activity and thus vegetation cover, showed roughly the same seasonal pattern as relative humidity and was invariably at a minimum at the end of the hot dry season (Fig 2B).

When days were viewed singly, the numbers of hours suitable for feeding varied between 1 and 13 hours (Fig E in S1 Appendix). When averaged by month the value varied between about 7 and 13 (Fig 2A). Mean hours suitable for feeding were longest in January and February (11 hours) and shortest in September – November (8, 6.5 and 7 hours, respectively). For all other months the mean was 9–10 hours (Fig F in S1 Appendix). Months with the lowest number of hours suitable for feeding were also those in which temperature and saturation deficit were highest, and relative humidity and NDVI were lowest (Fig 2).

### Seasonal variation in the durations of oogenesis, pregnancy and pupal period

**Fig 3:**
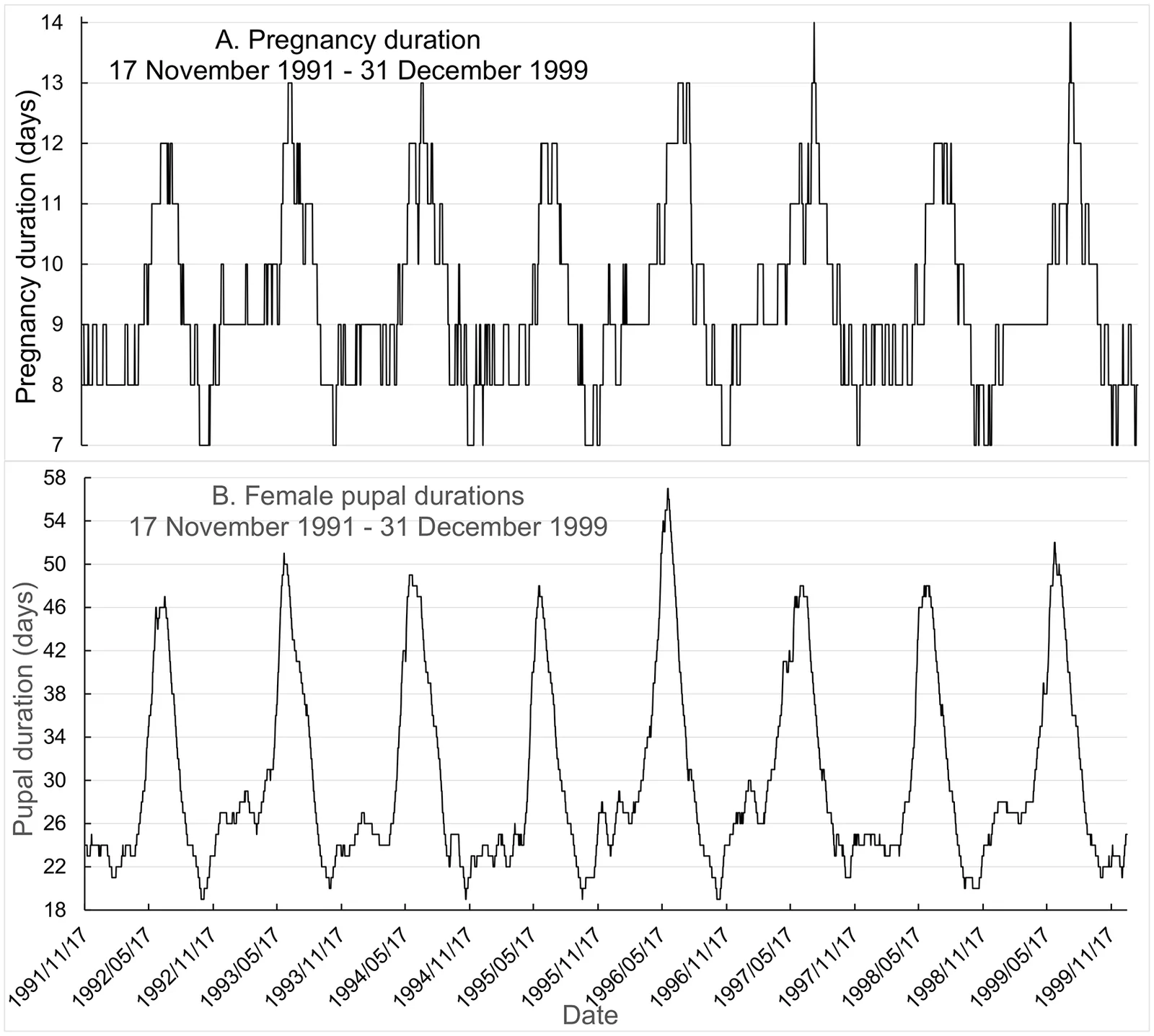
Estimated durations of pregnancy and female pupal duration. A. Pregnancy; B. Female pupal duration, assumed identical for *G. m. morsitans* and *G. pallidipes*, by day of larviposition in typical larviposition sites, at Rekomitjie, November 1991 – December 1999.

**Fig 4:**
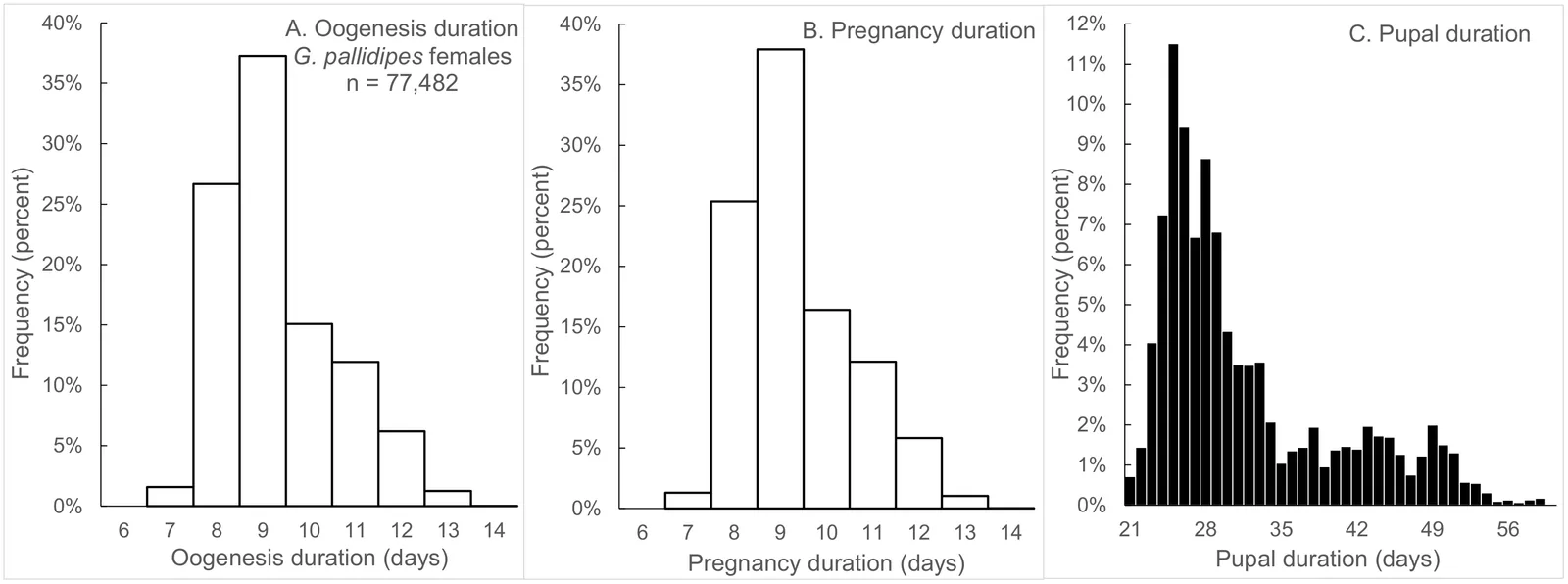
Durations of oogenesis, pregnancy and pupal life. A. Oogenesis (the time that the largest oocyte spends in the ovary); B. Pregnancy. C. Pupal life. All calculated from the estimated times of the starts and ends of oogenesis, pregnancy and pupal development of individual flies in ovarian categories *c* = 0-3. For Figs 4 and 5 we used flies for which there was a full life history but did not exclude flies if the wing length was missing; hence the slightly larger *n* than in Table 3, row 4.

Our life history estimates generated the durations of pregnancy and pupal life for all young flies (*c* < 4) starting either of these processes on any day between 17 November 1991 and the end of 1999. All durations were shortest in November and longest in July of each year (Fig 3). Over the period of analysis, pregnancy duration for *G. pallidipes* varied from 7 to 14 days (Fig 3A), but 84% of oogenesis and pregnancy durations were in the interval 8–11 days, inclusive (Fig 4A, 4B). Pupal durations, likewise, varied over the large range of 19–57 days, but 70% lay in the interval 23–33 days, inclusive (Fig 4C).

**Fig 5:**
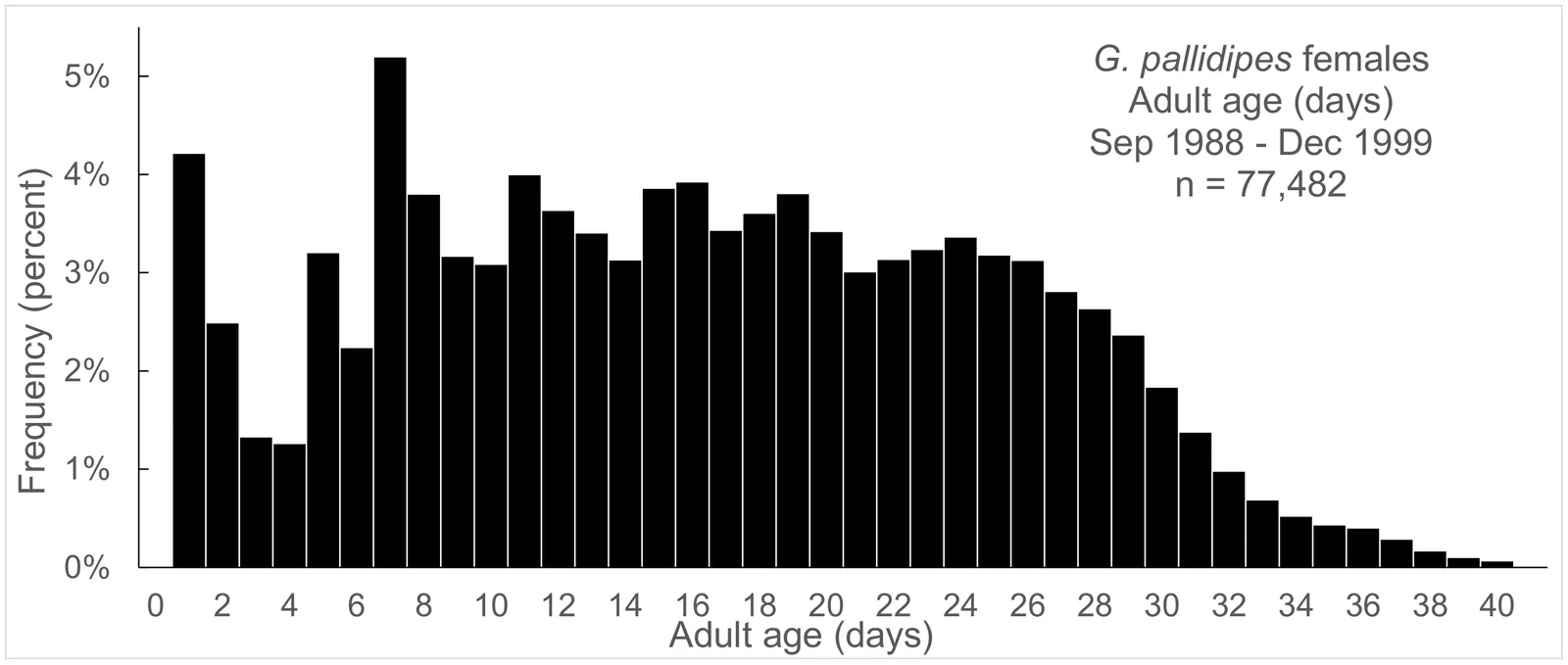
Distribution of ages of female *G. pallidipes.* All ages were estimated using ovarian dissection. Note that ages are only estimated for flies where *c* = 0-3.

The age distribution of all *G. pallidipes* females for which we had a life history shows an early peak of activity immediately following adult emergence (Fig 5) followed by a hiatus in catches until the age of 7–8 days – when females ovulate for the first time. Lower frequencies at ages 2–6 days may be attributed to reduced activity in flies that are using their first two blood meals to build thoracic flight muscle [4,5,39–41]. Frequencies change little for ages 8–26 days, then fall away because significant proportions of flies have ovulated for a fourth time: we could not confidently estimate their chronological age and did not, therefore, generate their life histories.

### Distributions of egg and wing lengths

Egg and wing lengths are both greater in *G. pallidipes* than *G. m. morsitans* but the difference is much less marked in eggs (Figs G, H in S1 Appendix). Samples were excluded from analyses if it seemed likely that the fly had been identified as the incorrect species, or if there had clearly been an error in the measure of the egg or wing length. This happened in <1% of wings measured, and <0.1% for eggs (Table A in S1 Appendix).

### Seasonal changes in egg and wing lengths

**Fig 6:**
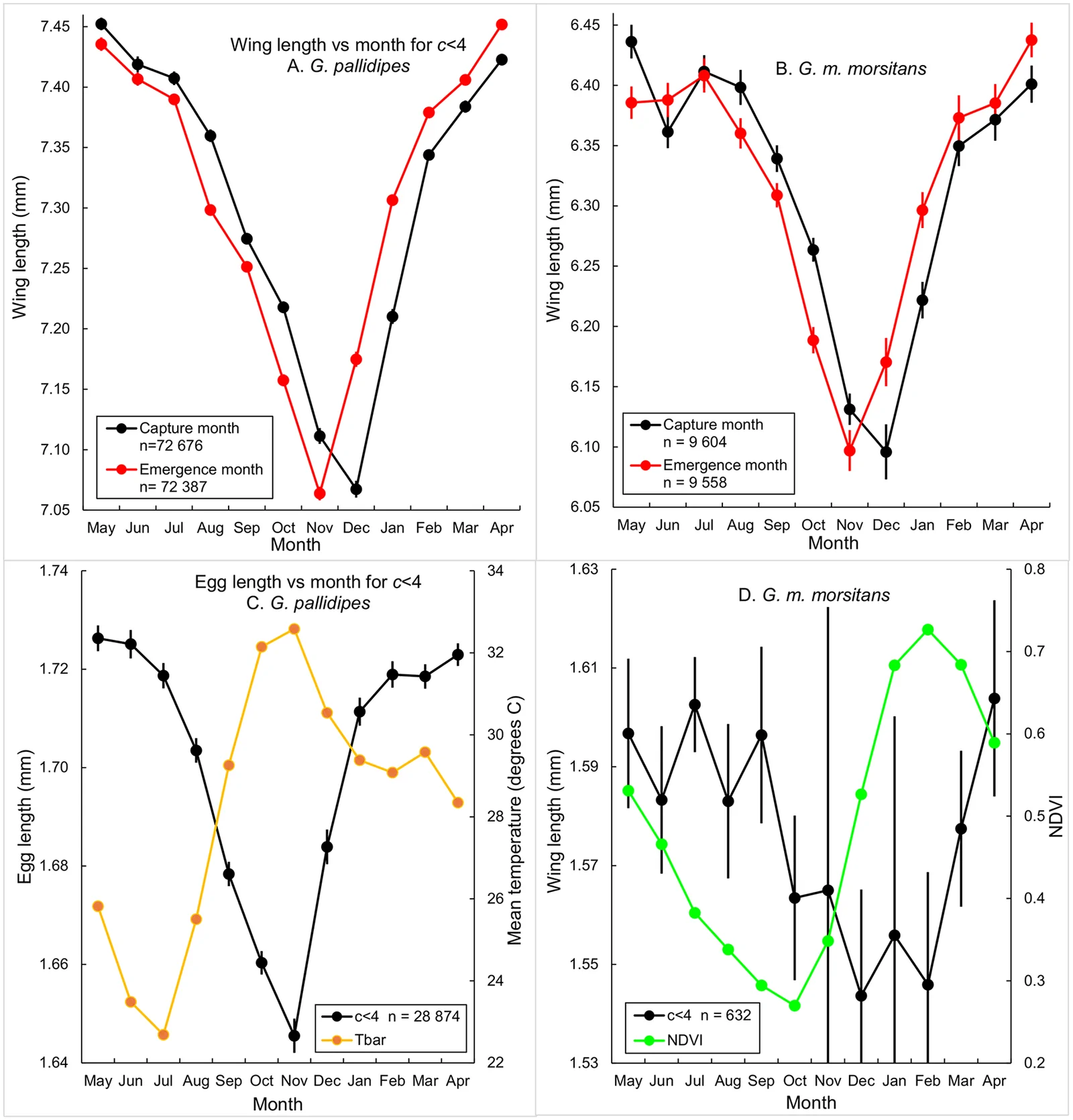
Mean wing and egg lengths. Mean wing and egg lengths of *G. pallidipes* (A, C) and *G. m. morsitans* (B, D) sampled at Rekomitjie between September 1988 and December 1999. Lengths are plotted as a function of the month of capture of the adult female, and wing lengths also against the month in which females were estimated to have emerged as adults. Catches are pooled over all years and restricted to females that had ovulated fewer than four times. In A and B, *n* is larger for capture month than for emergence month because, in 0.4% of cases, missing length measurements meant flies’ emergence dates could not be estimated. Sample sizes are slightly smaller than in Table 3, row 4, because here we exclude flies with minor abnormalities in ovarian dissection measurements. Samples sizes in C and D are smaller than in Table 3, row 7, because here we restrict the sample to flies where c < 4.

Mean egg and wing lengths of female *G. pallidipes* and *G. m. morsitans* pooled by month of capture, varied in a highly regular fashion with season – with a minimum in December at the end of the hot dry season and beginning of the rains (Fig 6). Thereafter, mean wing length increased rapidly, peaking in about April/May in the warm dry season, then declined again rapidly between August and December. There was a similar regularity in the changes with time in mean wing lengths of flies estimated to have emerged in each month – but the cycle of changes lagged by about a month behind the length changes for flies captured in that month. The same pattern of length changes was evident every year (Fig I in S1 Appendix).

**Fig 7:**
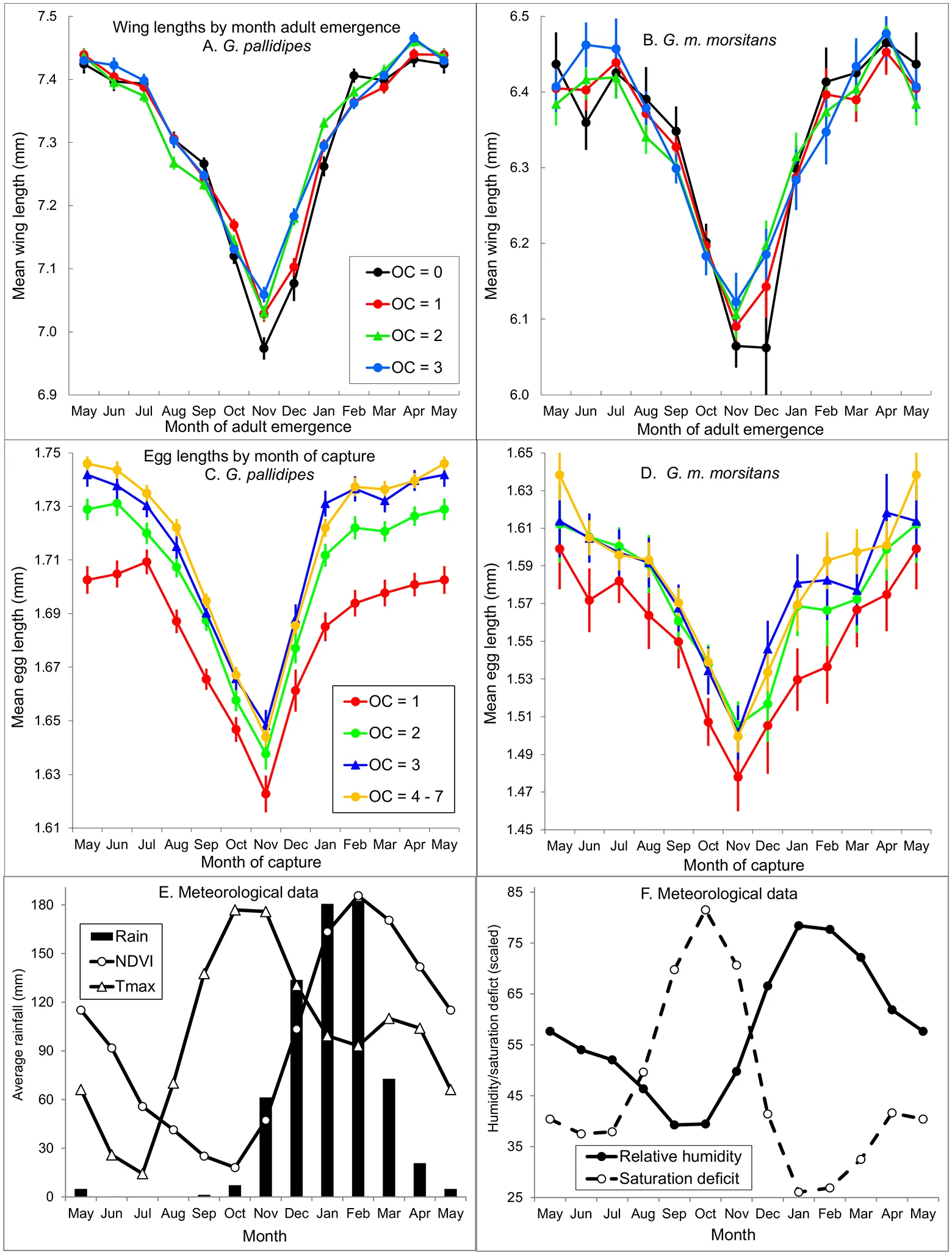
Mean wing and egg lengths. Mean wing and egg lengths for females of *G. pallidipes* (A, C) and *G. m. morsitans* (B, D). Eggs were pooled by the month of their mother’s capture; adults on the estimated month of their emergence, which could only be estimated for flies of ovarian category (OC) < 4. (E) Mean monthly values of mean daily temperature (*Tbar*), NDVI and rainfall. (D). Relative humidity and saturation deficit were available only from November 1991. NDVI and *Tbar* in (E) and saturation deficit in (F) were scaled to show relative timing of changes with season of all five variables. Minimum and maximum monthly values for the scaled variables were: NDVI (0.225, 0.664); *Tbar* (20.88, 29.84); saturation deficit (8.68, 27.17).

We pooled wing and egg lengths on year of capture, then estimated the mean lengths as a function of month and of ovarian age category (Fig 7). Mean wing lengths for both *G. pallidipes* and *G. m. morsitans*, of all ovarian categories, declined sharply between June and December, most particularly for flies where *c* = 0 (Fig 7A, 7B). For egg lengths, the picture was even clearer, with the eggs of youngest mothers (*c* = 1) being significantly shorter, in every month of the year, than eggs of all older flies (Fig 7C, 7D). The eggs of *G. pallidipes* female where *c* = 2 were also significantly shorter than for older flies, throughout the year. The difference was less marked for *G. m. morsitans*, reflecting in part the smaller sample sizes for this species. Age effects are seen more clearly in Fig J in S1 Appendix, where egg lengths are plotted against ovarian category for data stratified by month of maternal capture.

### Wing length as a function of age and of month of either adult emergence or of capture

#### Month of emergence as an adult.

Wing lengths for females of both species that emerged within the week prior to capture (*c* = 0), declined steadily from July – November, then increased rapidly until February, with smaller change between February and July (Fig 6A, 6B). Within most months of the year, mean wing length varied little with ovarian category *c*, consistent with the implicit assumption that the wing length of an individual adult tsetse does not vary with age. In November and December, however, mean length increased markedly with *c*. These changes can be seen more clearly when wing lengths of female *G. pallidipes* were plotted against *c*, for each month of the year in which the fly was estimated to have emerged as a teneral adult (Fig K in S1 Appendix).

The increases in wing length with age, during the hot season, are consistent with disproportionately high losses among small, young, flies in hot-dry months, so that mean sizes of surviving older flies were thereby increased. This accords with published evidence indicating selection against small individuals in several different tsetse species [8,10,15,42]. Previous authors all concluded, however, that losses of small flies were restricted to the teneral period, *i.e*., only in very young flies. By contrast, Fig K in S1 Appendix suggests that the size of flies emerging as adults in October through December continues to increase for some weeks after flies would have taken their first blood meal.

**Fig 8:**
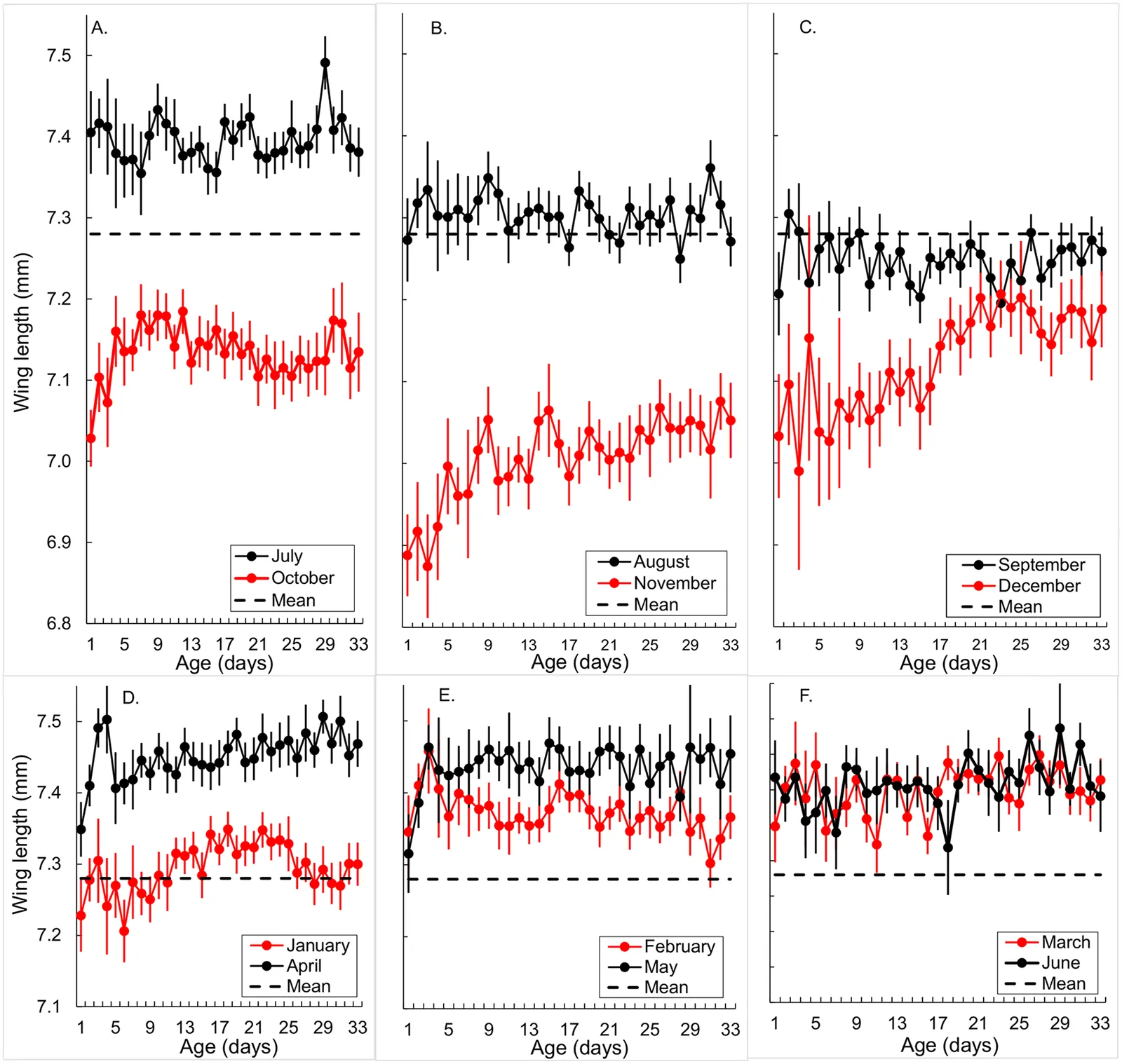
Mean wing length versus fly age and emergence date. Wing length in female *G. pallidipes* sampled at Rekomitjie from September 1988- December 1999, as a function of the flies’ daily ages and months in which the flies were estimated to have emerged as teneral adults. Vertical bars indicate 95% confidence intervals around daily age means. Wing length changes little with age in June-September but increases markedly in October-January – consistent with elevated mortality of small flies at the hottest times of the year. Horizontal dashed lines indicate the overall mean wing length, across all flies. Total sample size; 76,506. This is slightly less than the number in Table 3, row 4 because here we restrict the analysis to flies of a maximum estimated chronological age of 33 days.

We investigated the discrepancy further, using flies whose ages were estimated to the nearest day. In Fig 8A – 8C, note that from July through September wing lengths were getting shorter each month, but there was little change in wing length with age, within each month. In October and November, the wing lengths of the youngest flies continued to get progressively smaller (Fig 8A, 8B); but there was then a more sustained increase in wing length with age within each month, with the November increase continuing for more than three weeks. In December (Fig 8C), when the rains arrived and temperatures dropped, wing length started from a higher base in young flies, but there was still a sustained increase with age over the first three weeks of life. This trend was weakly sustained in January (Fig 8D); in February (Fig 8E), changes with age were restricted to flies in the first week of life. The same was true for April and May (Fig 8D, 8E); for March and June there was little change with age (Fig 8F).

#### Month of capture.

**Fig 9:**
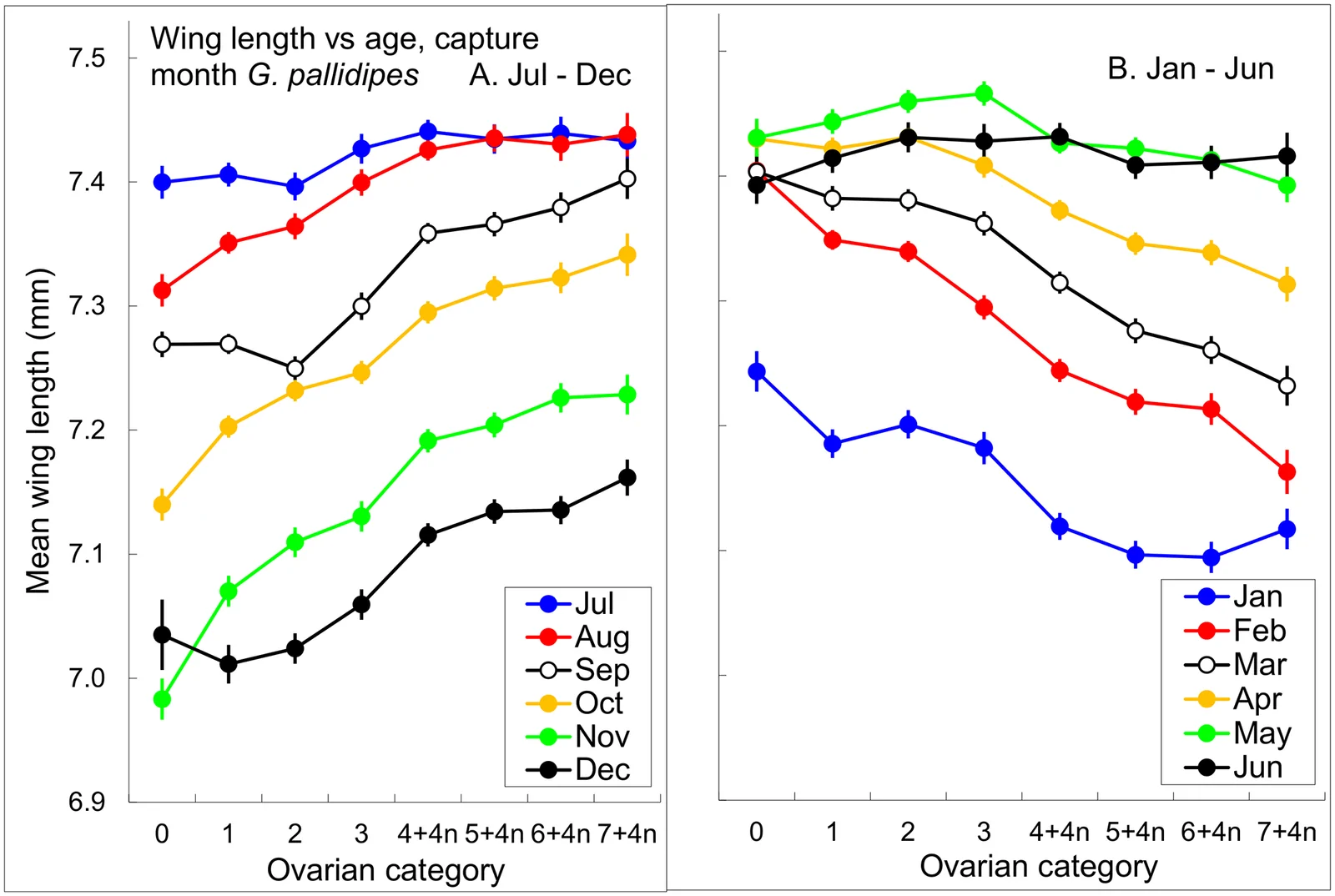
Mean wing length versus ovarian age and capture month. Wing length in female *G. pallidipes* sampled at Rekomitjie between August 1988 and December 1999, as a function of the ovarian age of the fly and the month in which the fly was captured. Total sample size; 151,269.

When flies were grouped with respect to their month of capture, rather than emergence, there were more striking age-related changes in wing length. From August to December, mean length increased sharply with ovarian age (Fig 9A), whereas in January through April, the mean length decreased with age (Fig 9B). These changes are readily understood as a natural consequence of the seasonal changes in the wing lengths of emerging adults. For example, the oldest flies among those sampled in December would have been deposited as larvae in the cool dry season, when the flies are biggest, while the youngest flies would have been deposited at the height of the hot dry season when they are smallest. Likewise, in February, the youngest flies will have been deposited in January and February, when relative humidity is highest and temperatures are declining (Fig 2) – whereas flies of ovarian ages 4 + 4*n* to 7 + 4*n* will have been deposited in September-November, when temperatures are highest and relative humidity lowest. Similar analyses carried out on the data available for *G. m. morsitans* provided a generally similar picture – based on much smaller samples size with, accordingly, greater variability (Fig L in S1 Appendix). The steady decrease in wing and egg lengths in the second half of each year coincides with the onset of the hot dry season in the Zambezi Valley and increases in length began with the onset of the rains (Fig 7E, 7F). These results raise the question of which meteorological features are best correlated with the observed changes in egg and wing length.

### Analysis of factors affecting egg length in *G. pallidipes* and *G. m. morsitans*

#### Individual flies.

Correlation matrices for egg length versus six environmental variables, plus maternal ovarian category and wing length, showed that egg length in both species was highly correlated with a variety of meteorological factors. These included temperature, relative humidity, saturation deficit, NDVI, rainfall and the number of hours per day suitable for feeding activity, all averaged over the estimated period of time when the egg had been developing as an oocyte in its mother’s ovary (Tables B, C in S1 Appendix). For both species, egg length was significantly reduced for *c* = 1 relative to older flies (Tables D, E in S1 Appendix), in accord with the results in Fig 7C, 7D. We could detect, however, neither a progressive increase in egg length with maternal age, nor any indication that egg size decreases in the oldest flies (Fig 7C, 7D).

There were strong correlations between various meteorological variables: NDVI, relative humidity and rainfall all provided measures of moisture; saturation deficit provided a combined measure of heat and dryness; and the number of hours suitable for tsetse feeding behavior each day is a function of temperature. These high correlations would confuse any interpretation of multiple regression models involving all candidate explanatory variables. Moreover, full-model regressions of egg length for individual flies accounted for only 18% of the variance for *G. pallidipes* and 21% for *G. m. morsitans* (Tables D, E in S1 Appendix). We therefore carried out all further regression modelling using mean lengths of eggs, and of corresponding environmental predictor variables, averaged weekly for *G. pallidipes* and monthly for *G. m. morsitans*.

### Flies pooled over weeks/months

Pooling was based on the estimated date when oogenesis was completed. Weekly averaging provided 525 weeks of *G. pallidipes* averages, for the full period 1988–1999, 479 of which included five or more eggs in their averaging, weeks with fewer than 5 eggs having been excluded from the analysis. Models were developed using 341 of these weeks which lay in the period starting in week 47 (November 1991) – the first week for which weekly logger data were available. Regression using all candidate predictors accounted for 77% of the variance (Table F in S1 Appendix). Note the high *t*-value for mean maternal wing length, despite it being averaged over many flies.

Using the analogous approach for monthly-pooled averages for *G. m. morsitans* data provided 124 months for the full period (1988–99), 100 of which were based on a minimum of five eggs: models were based on 65 of these months, starting in December 1991 when the logger data became available. Regression using all candidate predictors accounted for 66% of the variance and, as with *G. pallidipes*, maternal wing length (*wlmm*) had the highest *t*-value (Table G in S1 Appendix).

**Fig 10:**
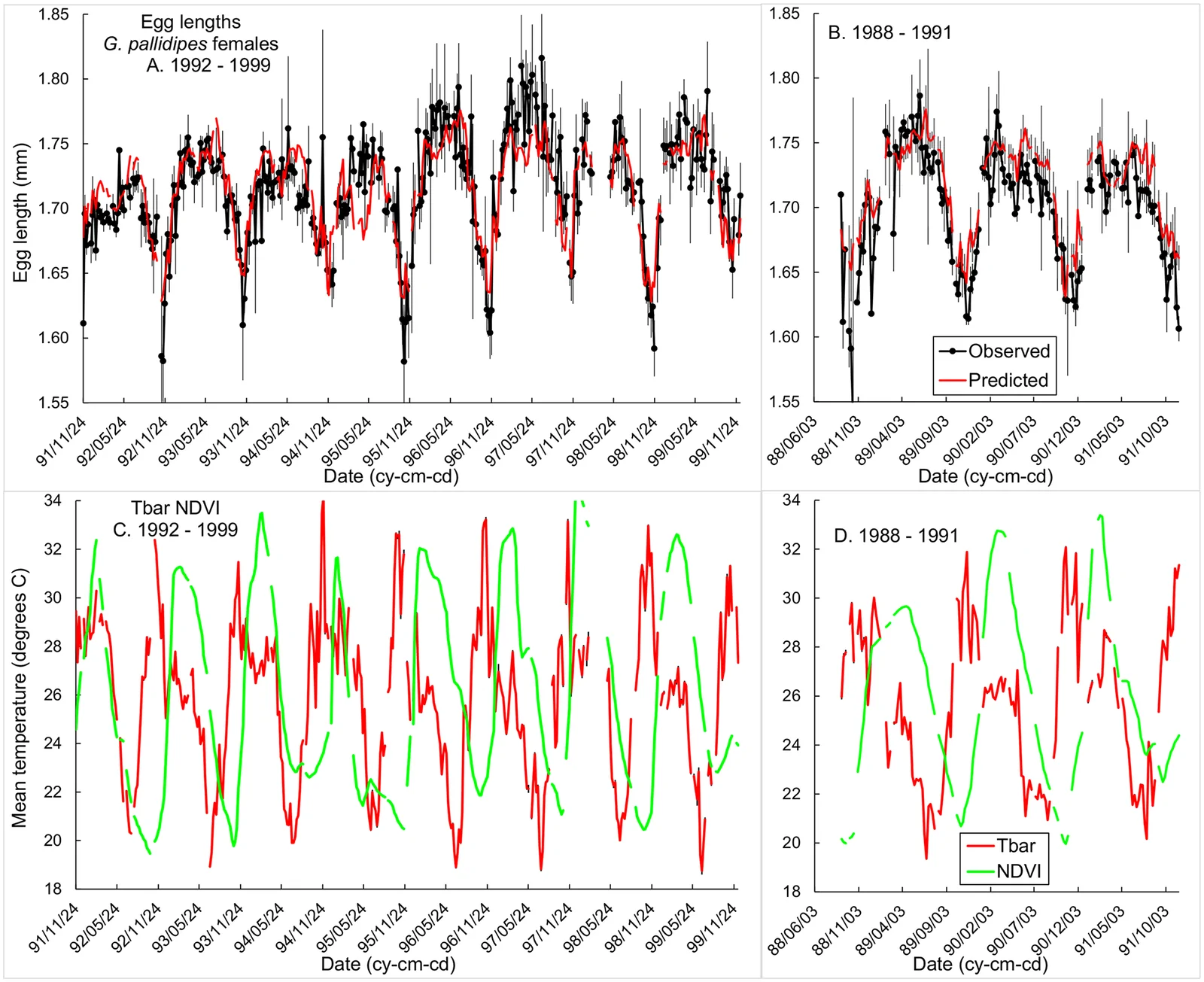
Egg lengths of *G. pallidipes* sampled at Rekomitjie. Observed weekly mean egg lengths, with 95% confidence intervals, for flies pooled weekly based on the end of oogenesis, for A, December 1991 – December 1999: B, November 1988 – November 1991. Predicted lengths in A result from fitting the model shown in Table 4A. Predicted weekly mean lengths in B are true predictions from the model that was fitted to the data in A. C, D. Weekly mean temperatures and NDVI calculated over the period of oogenesis leading to the production of eggs in A and B, respectively.

**Fig 11:**
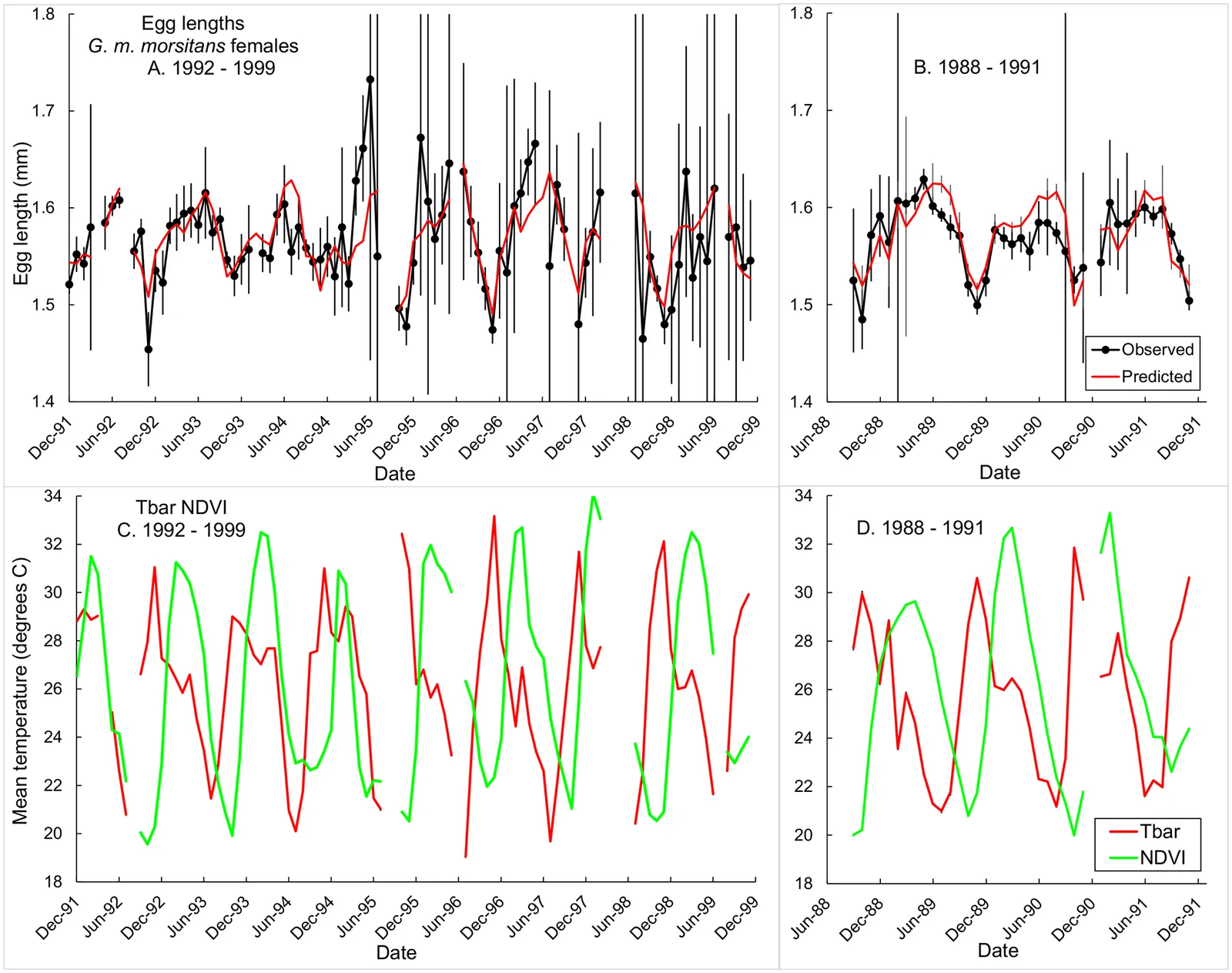
Egg lengths of *G. m. morsitans* sampled at Rekomitjie. Observed monthly mean egg lengths, with 95% confidence intervals, pooled monthly based on the end of oogenesis, for **A.** December 1991 – December 1999: **B.** November 1988 – November 1991. Predicted lengths in A result from fitting the model shown in Table 4B. Predicted monthly mean lengths in B are true predictions from the model that was fitted to the data in **A.** C, D. Temperatures and NDVI calculated over the period of oogenesis leading to the production of eggs in A and B, respectively.

To make true predictions of egg length, for 1988–91, we did not include mean *wlmm*, from our sampled tsetse data from that period, as a predictor variable; if we use tsetse data from that period, then predicting egg length is moot - we could simply obtain egg lengths directly from the data. Instead, we set aside the observed egg lengths from 1988-91 as a check on values from our true predictions. Thus, only the three variables *Tbar*, *ndvi*, and *rain* are available for driving predictions during 1988–91. Stepwise regression was run, based on the AIC criterion, to select the best model from the above three candidates and showed that, for both species, mean rainfall could be dropped with no significant change in AIC. Accordingly, the models used for prediction of egg lengths in the period 1988 – 1991 are based only on mean temperature and NDVI. Results of the final regression models are shown in Table 4A, 4B and were used in the construction of Figs 10 and 11. For *G. pallidipes*, the simpler regression accounted for 69% of the variance in egg length, less than that for the full model including maternal wing length and additional environmental factors. Nonetheless, the model produced a good fit both to the data collected after November 1991 (Fig 10A), and it also provided true predictions for 1988–91 that matched well the observed mean egg lengths from that period (Fig 10B).

**Table 4:** Regression of egg length (*ulmm*) in tsetse as a function of mean temperature (*Tbar.*wkmn) and NDVI (*ndvi.*wkmn).

A. *G. pallidipes* Data pooled over weekly periods as described in the text. Call: lm(formula = *ulmm*.wkmn ~ *Tbar*.wkmn + *ndvi*.wkmn, data = modl.dat)					
Coefficients	Estimate		Std. Error	t value	Pr(>|t|)
(Intercept)	1.871		0.0110	170.5	<2e-16 ***
*Tbar*.wkmn	-0.00831		0.000397	-20.9	<2e-16 ***
*ndvi*.wkmn	0.136		0.00792	17.2	<2e-16 ***
Residual standard error: 0.0306 on 62 degrees of freedom. Multiple R^2^: 0.542, Adjusted R^2^: 0.527; *F*-statistic: 36.7 on 2 and 62 DF, *p*-value: 3.056e-11. B. *G. m. morsitans* Data pooled over monthly periods as described in the text. Call: lm(formula = *ulmm*.*momn* ~ *Tbar*.*momn* + *ndvi*.*momn*, data = modl.dat)					
Coefficients:		Estimate	Std. Error	t value	Pr(>|t|)
(Intercept)		1.825	0.0383	47.7	< 2e-16 ***
*Tbar*.*momn*		-0.0105	0.00132	-7.97	4.41e-11 ***
*ndvi*.*momn*		0.0471	0.0234	2.02	0.048 *

Residual standard error: 0.0238 on 338 degrees of freedom.Multiple R^2^: 0.686; Adjusted R^2^: 0.684; *F*-statistic: 368.9 on 2 and 338 DF, *p*-value: < 2.2e-16. In all Tables the following codes indicate probability levels: < 0.001 ***; < 0.01 **; < 0.05 *.

Given the much smaller sample sizes for *G. m. morsitans*, the variability in the observed pooled egg lengths was much greater than for *G. pallidipes*. Nonetheless, there is good correspondence between the observed and predicted egg lengths, in both the model fitting and model prediction periods (Fig 11). For both species, minimum egg lengths were closely aligned with peaks in the mean temperature, increasing rapidly thereafter as NDVI values increased (Figs 10, 11C and 11D).

## Analysis of factors affecting wing length in *G. pallidipes* and *G. m. morsitans*

### A. *G. pallidipes*

#### Individual flies.

We modelled the effect of mean meteorological conditions, prevailing at the time a female tsetse was developing in its mother’s abdomen, on the wing length of the resulting adult.

Product-moment correlations showed that mean saturation deficit, relative humidity, and NDVI were equally strongly correlated with wing lengths. However, linear regression of length on all 6 mean meteorological variables explained only 24% of the variance in wing lengths of the 48,795 individual *G. pallidipes* (Tables H, I in S1 Appendix). Accordingly, further regression modelling used only the averages from the weekly pooled groups of flies.

#### Weekly pooling.

For *G. pallidipes* all further analyses involved data pooled over weeks. The matrix of correlations between mean wing length and six predictor variables showed that there were strong correlations between mean wing length, saturation deficit, relative humidity, NDVI and the number of hours available for feeding (Table J in S1 Appendix). For the pooled data, linear regression, using all six available independent variables, accounted for 71% of the variance in wing length (Table K in S1 Appendix). Note that temperature (*Tbar*) and relative humidity (*RHbar*) are high correlated with saturation deficit *(SDbar*), and removing the first two of these variables from the regression did not affect the R^2^ value. The full-model regression could not, however, be used to predict wing lengths prior to November 1991 – since saturation deficit and the numbers of hours available for feeding (*HrsFeed*) were not measured during that period. Accordingly, we modelled wing length as a function only of temperature, NDVI and rain. This regression accounted for 64% of the variance in wing length (Table 5). Stepwise selection based on AIC showed that all three of these variables contributed predictive value.

**Table 5:** Regression of mean weekly wing length (*wlmm*) in *G. pallidipes* as a function of the mean temperature (*Tbar.*wkmn), NDVI (*ndvi.*wkmn) and rain (*rain.*wkmn). Wing lengths are the mean values for flies caught in consecutive one-week (7-day) periods; temperatures, NDVI and rain are the estimated mean values of these variables over the period when the captured flies were developing in the ovaries and uteri of their mothers.

Coefficients:	Estimate	Std. Error	t value	Pr(>|t|)
(Intercept)	7.564	0.0482	157.0	<2e-16 ***
*Tbar.*wkmn	-0.02267	0.00173	-13.1	<2e-16 ***
*ndvi.*wkmn	0.7314	0.0362	20.2	<2e-16 ***
*rain.*wkmn	0.005194	0.00189	2.75	0.00621 **

Residual standard error: 0.108 on 410 degrees of freedom

Multiple R^2^: 0.635. Adjusted R^2^: 0.633

F-statistic: 238.1 on 3 and 410 DF; p-value: < 2.2e-16. AIC = -1840.2

#### Observed and predicted wing length November 1991 – December 1999.

**Fig 12:**
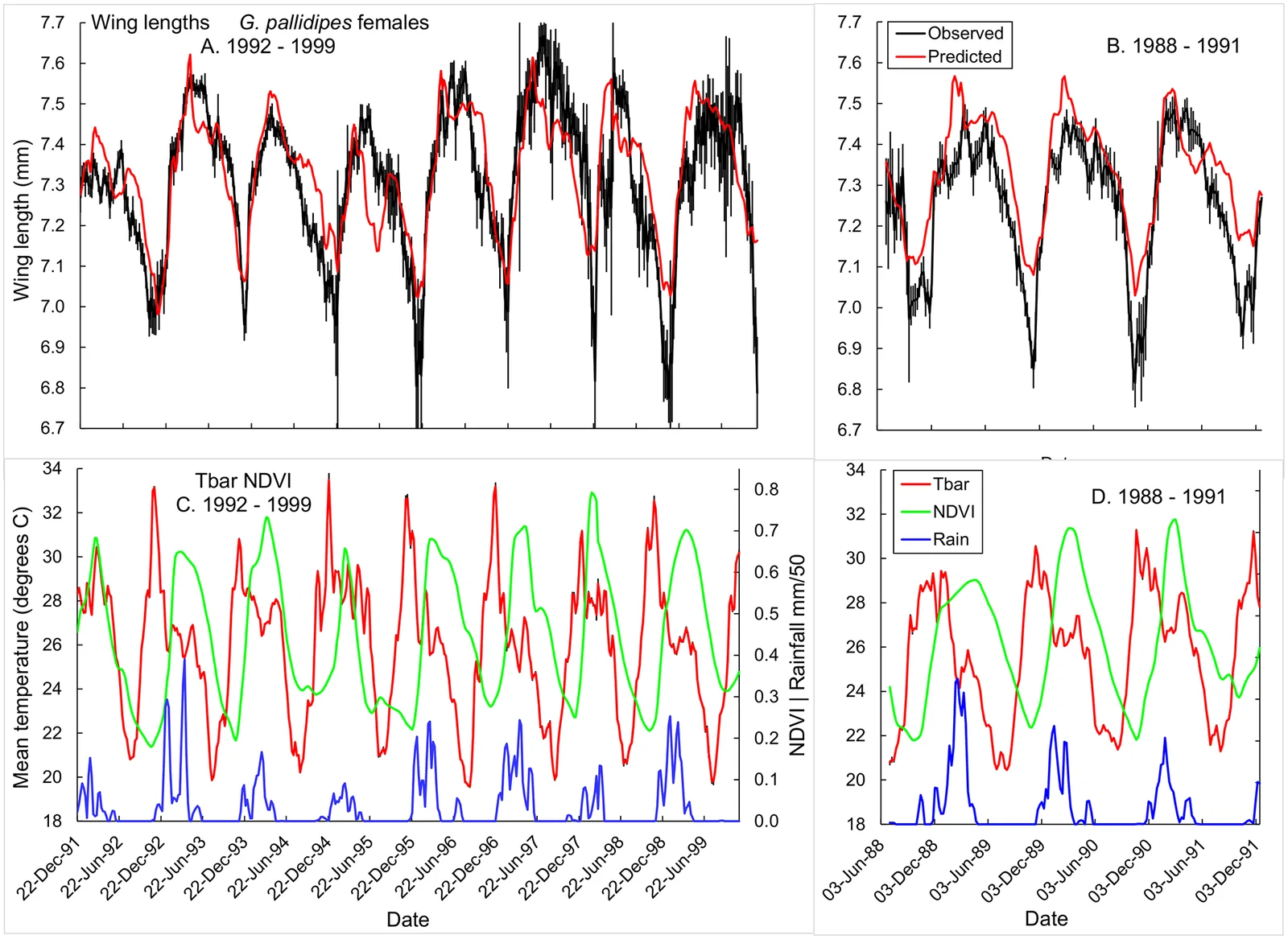
*G. pallidipes* female wing lengths versus larviposition date. Observed weekly mean wing lengths of *G. pallidipes* females at capture (black lines, with 95% confidence intervals) plotted against the week of their larviposition. Red lines show lengths predicted by the model detailed in Table 5. (A) Flies captured 17 Nov 1991 - 31 Dec 1999; (B) 13 Sep 1988 - 16 Nov 1988. Red, green and blue lines in Fig 12C and 12D show corresponding weekly mean temperatures, NDVI and rainfall, respectively, averaged over the periods of oogenesis and pregnancy – *i.e*., while the fly was developing within its mother’s ovaries and uterus, respectively.

A plot of observed mean weekly wing length, overlaid with model predictions driven by mean temperature, NDVI and rainfall, showed good agreement between the two time series (Fig 12). Weekly mean wing lengths at time of capture were consistently synchronized with annual cycles in NDVI experienced during fly development (Fig 12A, 12C). NDVI increased rapidly after the onset of the rains in December and declined steadily when the rains stopped in about March. Mean wing size increased rapidly with increasing NDVI during the last quarter of each year, peaking shortly after the peak NDVI in January-February. Subsequently, an annual decline in mean wing length usually continued to follow NDVI, with a lag of 1–2 months. There was an exception in 1999, when wing length remained high throughout most of the year. Peak annual temperatures experienced by flies during their development coincided with minimum length at time of capture (Fig 12A, 12C), although the annual coincidence between lower temperatures and greater size was less consistent.

#### Observed and predicted wing length September 1988 – November 1991.

Having used flies captured after 16 November 1991 to develop a predictive model for wing length as a function of *Tbar, ndvi* and *rain*, we applied the predictions to the period between the start of the study in 1988 and 16 November 1991. We did no further fitting to the earlier data; we simply used the temperature, NDVI and rainfall data during that period to make true predictions of the weekly mean wing lengths. Fig 12B shows that there was good agreement with the observed values.

### B. *G. m. morsitans*

#### Individual flies.

For this species we had full histories for 10,616 individual females, only 14% of the number available for *G. pallidipes*. As expected, full-model regressions of wing length against all 6 meteorological variables, carried out at the level of individual flies, had an *R*^2^ value of only 0.19 (Tables L, M in S1 Appendix). Weekly averaging of flies resulted in 25% of the 387 weeks in 1991–99 having samples of 5 or fewer flies, and 50% of the weeks having 10 or fewer. Such small sample sizes would have generated noisy weekly means of wing length, with wide confidence intervals. We therefore carried out further regression modelling only on the means from monthly groups of flies.

#### Monthly pooling.

Flies were assigned to monthly groups, based on their estimated larviposition date. After monthly averaging, only 5 of 92 months had fewer than 10 flies. We ran two regression models, based on monthly averages for the 1991–99 period. For the full model, using all meteorological variables, *R*^2^ = 0.66 – a good result, given the extensive averaging of the data (Tables N, O in S1 Appendix).

#### Observed and predicted wing length November 1991 – December 1999.

**Fig 13:**
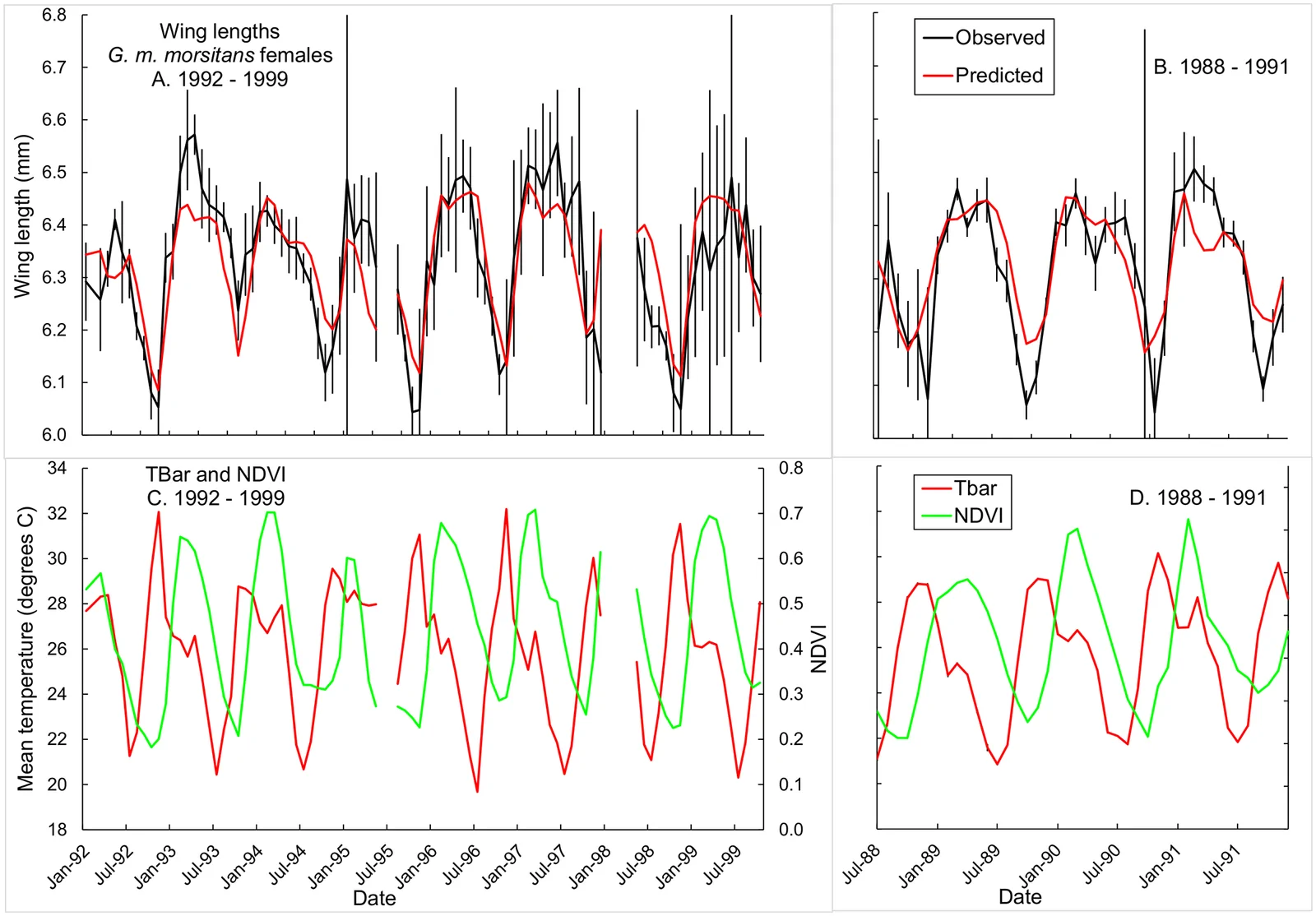
*G. m. morsitans* female wing lengths versus larviposition date. Monthly mean wing lengths of adult *G. m. morsitans* females at capture (black lines, with 95% confidence intervals), plotted against the month of their larviposition. Solid red line shows the lengths predicted by the model detailed in Table 6.

We then used a model based only on *NDVI* and *Tbar* (Table 6) – variables that were also used for the final *G. pallidipes* model. For this simpler model, *R*^2^ was 0.61, slightly less than for the full model. Based on model AIC, mean rainfall did not add any predictive value to the simpler model. Both predictors were significant, with coefficient signs the same as the similar model for *G. pallidipes*. NDVI and temperature were thus the primary drivers for both species, with qualitatively similar effects – despite the two species being modelled on different time scales. The model provided good fits to the monthly averaged wing length data for December 1991 – December 1999 (Fig 13) and, as with *G. pallidipes*, changes in wing length were quite closely synchronized with those in NDVI.

**Table 6:** Mean monthly wing lengths of female *G. m. morsitans* regressed on monthly means of mean daily temperature (*Tbar*) and *ndvi.*

Call: lm(= length.momn ~ *Tbar*.momn + *ndvi*.momn, data = momn.log)					
Residuals:	Minimum	1Q	Median	3Q	Max
Coefficients:	Estimate	Std. Error	t value	Pr(>|t|)	
(Intercept)	6.594	8.58e-02	76.82	<2e-16 ***	
*Tbar.momn*	-0.019	3.06e-03	-6.20	2.1e-08***	
*ndvi.momn*	0.528	5.80 e-02	9.05	4.75e-14***	

Residual standard error: 0.08386 on 84 degrees of freedom. Multiple R-squared: 0.610, Adjusted R-squared: 0.601. F-statistic: 65.7 on 2 and 84 DF, p-value: < 2.2e-16

#### Observed and predicted wing length November 1991 – December 1999.

Following the same procedure as for the *G. pallidipes* we see that the model (Table 6) fitted to data from November 1991 – December 1999 (Fig 13A), also provided – without further fitting – good true predictions for the 1988–91 period (Fig 13B).

## Discussion

Insect developmental rates have traditionally been used to predict future development and emergence dates of insect crop-pests – generally with the aim of optimizing the timing of insecticide application; [43–45]. We reversed this procedure, assigning to field-sampled female tsetse a timetable of life stages in the flies’ pasts. We then assessed effects of meteorological conditions, at any chosen period of a fly’s life history, on the state of adult flies at the time of their capture. Published estimates of development rates in different tsetse life stages, and detailed ovarian dissection data, facilitate accurate age estimates [15,23,46]. We analyzed an order of magnitude more flies than in previous studies of this type (*cf.* Table 3 and Fig M in S1 Appendix).

### Predicting future changes in egg and wing lengths

Good fits to egg and wing length data were obtained using weekly or monthly averages of fly sizes and regressing these mean lengths against time-averaged meteorological conditions prevalent during oogenesis and pregnancy. The fitted equations, based only on temperature and NDVI, provided good predictions of mean egg and wing lengths collected in earlier periods – without further fitting to those data. We could use the fitted equation to make true predictions of how fly size will change in the future, thereby providing important information on the expected effects of changing climate on tsetse biology.

### What drives size changes in tsetse?

We find the size of an adult tsetse sampled in the field is strongly correlated with measures of heat and moisture (temperature, relative humidity, saturation deficit and NDVI) prevailing when that fly was developing in its mother’s ovaries and uterus. We still cannot, however, see any direct causal link between size and any of the above independent variables. Instead, we suggest meteorological factors often have indirect effects on tsetse size, mortality and population density. Thus, even at temperatures that should not cause immediate direct harm to adult female tsetse, increasing temperatures mean increasing metabolic rates and elevated mortality due to the increased risks associated with more frequent feeding [47,48]. These risks are amplified for temperatures >32°C, when tsetse spend significant portions of the day in dark “refuges” [20]. There is then less time per day available for feeding – and elevated temperatures lead to yet higher metabolic rates, increased feeding rates, reduced pregnancy duration and thus less time available to provision the developing larva *in utero* [24,49,50]. The resulting increases in adult mortality will be accompanied by the production of smaller pupae and teneral adults in the next generation [22,32,51,52]

Changes in tsetse size and mortality are thus seen to be closely related. Indeed, a Kenyan study found that the size of adult female *G. pallidipes* emerging in the field was correlated with density-independent mortality, acting on the adult female population two months previously [15]. It was even suggested that offspring size might be used as an index of maternal mortality, which is difficult to measure directly in the field [21,29,53].

Observed declines with increasing temperature in the lengths of eggs, and of wing lengths in the resulting adults, are thus to be expected. Declining fly size brings a further problem, however, because there is severe selection at the hottest times of the year against the smallest tsetse, particularly because they also have relatively less fat than larger flies [42]. When the rains start, in December, temperatures decrease, humidity and NDVI levels increase, rates of vegetative growth are greatly accelerated, and many host species give birth in the following months. The previously declining trends in fly size are thereby all reversed; females produce larger eggs, larvae and, thereby, offspring (Fig 6A, 6B). Moreover, the rate of increase in the mean fly size accelerates because, between October and January, small flies are strongly selected against (Fig 8). Contrary to the findings of earlier studies, we show that this selection extends for some weeks after the teneral period. The larger surviving flies produce larger pupae (Table 6) and the larger adult daughters produce larger eggs (Table 4). The regular annual cycle in egg lengths may then be seen, largely, as a secondary result of changes in wing length: egg length is positively correlated with maternal size, though it is also a function of the conditions the adult female experiences during oogenesis.

Studies in West Africa found that tsetse distribution, population density, adult mortality, adult size, and seasonal changes in incidence of human trypanosomiasis, were all highly correlated with NDVI [14,54–57]. Again, no causal link was suggested, and it is unclear how NDVI could, for example, directly impact mortality. In these studies, the Moran curve approach [58] was used to estimate density-independent mortality per generation [59]; mortality was thus partitioned in some unknown manner between different life stages. For time series of population estimates of female *G. m. morsitans* on Antelope Island, Zimbabwe, Moran curve mortality was more highly correlated with saturation deficit than temperature [51,60]. This accords with the West African studies, since saturation deficit correlates significantly with NDVI. These results are consistent with measures of dryness, which are correlated with NDVI, having their greatest direct effect on immature stages of tsetse, particularly pupae and tenerals.

Adult mortality for the same flies was more highly correlated with temperature than with relative humidity or saturation deficit, which could indirectly affect fly size (see above). There could, however, also be indirect effects of NDVI on adult mortality and fly size. Large ungulate biomass is strongly correlated with rainfall [61], which is itself correlated with NDVI. As host density declines it will become increasingly difficult for tsetse to locate an animal to feed on, thereby increasing feeding related mortality. This was well illustrated by a game destruction exercise carried out in western Zimbabwe, where a 50% reduction in the game population led to a 95% reduction in the numbers of flies caught on fly rounds, implying significant increases in fly mortality [62]. Reduced feeding opportunity would likewise have reduced resources available for reproduction, leading to the production of smaller offspring. Analogous declines in host density occur naturally each year at Rekomitjie Research Station. The rapid decline in NDVI, following the onset of the dry season at Rekomitjie, reflects not only reductions in growth of green plants – but also the rapid consumption of that green vegetation by the herbivores that are also the hosts for tsetse. As the vegetation disappears, hosts may be expected to move in search of greener pastures, reducing the probability of a tsetse encountering a host.

### Limitations of our study

#### Sources of error.

The large proportion of unexplained variation, in regressions of individual fly sizes on meteorological variables, was due to several sources of uncertainty: these are summarized below and discussed more fully in Text A in S1 Appendix). (i) Measurement error: regression analysis demonstrated significant differences between egg length measurements made by five dissectors using eight different microscopes (Table P in S1 Appendix). (ii) Fly size exhibits remarkably small absolute variation, limiting the accuracy of our measurement (Fig N in S1 Appendix). (iii) All development rates were assumed identical for the two species, independent of fly size, and to be measured without error. While temperature, the driver of the rates, was accurately measured at a weather station, tsetse will generally be experiencing a different temperature [20,63]. Violations of these assumptions, along with age estimation errors, will lead to errors of a few to several days, in the timing of egg and wing developmental periods. Wetness, greenness and temperature change slowly on a longer timescale, however, perhaps explaining why we were so much more successful with modelling pooled data. (iv) Averaging fly attributes over weeks or months meant that maternal attributes, such as size and ovarian age, could not be linked to individual daughters, thus inhibiting the understanding of maternal effects. (v) Difficulties in estimating chronological ages [64]. We were unable to assign an age to 20–30% of the dissected flies, because they had ovulated more than three times. Our methodology could, however, be used in future to assess and calibrate new approaches to the problem of estimating age in field-sampled insects.

### Relevance of our study

Knowledge of the chronological ages of individual insects sampled in the field is pivotal in many areas associated with the study and control of insect vectors of disease: population dynamics and ecology, including age-dependent estimates of mortality and fecundity, age-dependent incidence and prevalence of infection and, thus, of vectorial capacity, and the assessment and improvement of vector and disease programs. Improved understanding of the causal links between fly size and mortality, and factors such as temperature, NDVI and host availability, facilitates improved predictions of how tsetse population density and distribution – and thus risk of trypanosome infection – varies with changes in environmental factors such as climate and land use. This knowledge aids the proactive identification of areas to be prioritized for monitoring and control efforts, thereby improving the cost-efficacy of tsetse and trypanosomiasis control.

Since our study appears to be unmatched in its scope by published work on tsetse, or any other fly, it serves as a model for similar work that could be carried out on other insect vectors of disease. It is now more than 75 years since Detinova published her groundbreaking study on the ovarian dissection of mosquitoes [65,66], serving as a model for the development of analogous techniques for other insects – applied to tsetse by Saunders [67] and refined by Challier [33]. But the technique has never been used successfully in field studies of mosquitoes, and deﬁning the age of a wild-caught mosquito remains a challenging, unreliable process [68]. If, however, ovarian dissection and new technologies could be combined, as we have suggested for tsetse, it might yet be possible to determine accurately the chronological ages of individual mosquitoes.

## Conclusions

We have developed a novel methodology for mapping a complete life history of field-collected female tsetse of ages up to *ca*. 35 days. This allowed us to demonstrate the effect, on a fly’s egg and wing lengths, of the meteorological conditions experienced by her mother while the fly was developing in her mother’s ovaries and uterus. We demonstrate the central importance of NDVI and the highly correlated humidity, and temperature, in determining fly size – and show that selection against under-sized individuals in the hot-dry season continues for some weeks after emergence rather than being restricted to mortality in teneral adults, as previously thought. Most importantly, we propose a mechanism for causal links between tsetse size and meteorological factors, which envisages indirect effects of measures of dryness and heat on the general well-being of tsetse populations via effects on the ease and safety with which the flies can feed.

## Supporting information

S1 Appendix Supporting Information for: Determination of size in tsetse. (PDF)

S1 Data Meteorological data for Rekomitjie Research Station. (XLSX)

S2 Data Life histories for individual female G pallidipes and G m morsitans. (XLSX)
