# Supplementary material for: Creating complete life histories of individual female tsetse (Glossina spp) to study the effects of meteorological conditions on fly size in Zimbabwe
Source: PLoS Negl Trop Dis. 2026 Aug 19;20(8):e0014120. doi: 10.1371/journal.pntd.0014120 (PMC13521475; doi:10.1371/journal.pntd.0014120)
Supplement: Supplementary file 1 [file pntd.0014120.s001.pdf]

S1 Appendix

Supporting Information for:

Creating complete life histories of individual female tsetse (*Glossina* spp) to study the effects of meteorological conditions on fly size in Zimbabwe

John W. Hargrove[1], Faikah Bruce[1], John Van Sickle[2]

[1] South African Centre for Epidemiological Modelling and Analysis (SACEMA), Centre for Epidemic Response and Innovation (CERI), School for Data Science and Computational Thinking, Stellenbosch University, Stellenbosch, South Africa

[2](Retired) Department of Fisheries, Wildlife, & Conservation Sciences, Oregon State University, Corvallis, Oregon, USA

Interpretation of ovarian dissections

Ovarian dissection: estimation of fly age

Dissection of tsetse ovaries allows us to assign each fly to an ovarian category, indicating the number of times it has ovulated (Challier, 1965). **Fig A** shows the disposition of the four oocytes within the paired ovaries at different stages of the life of a female tsetse fly. In the normal fly, oocytes develop and are ovulated in the strict order: right inner (labelled A), left inner (C), right outer (B) and left outer (D).

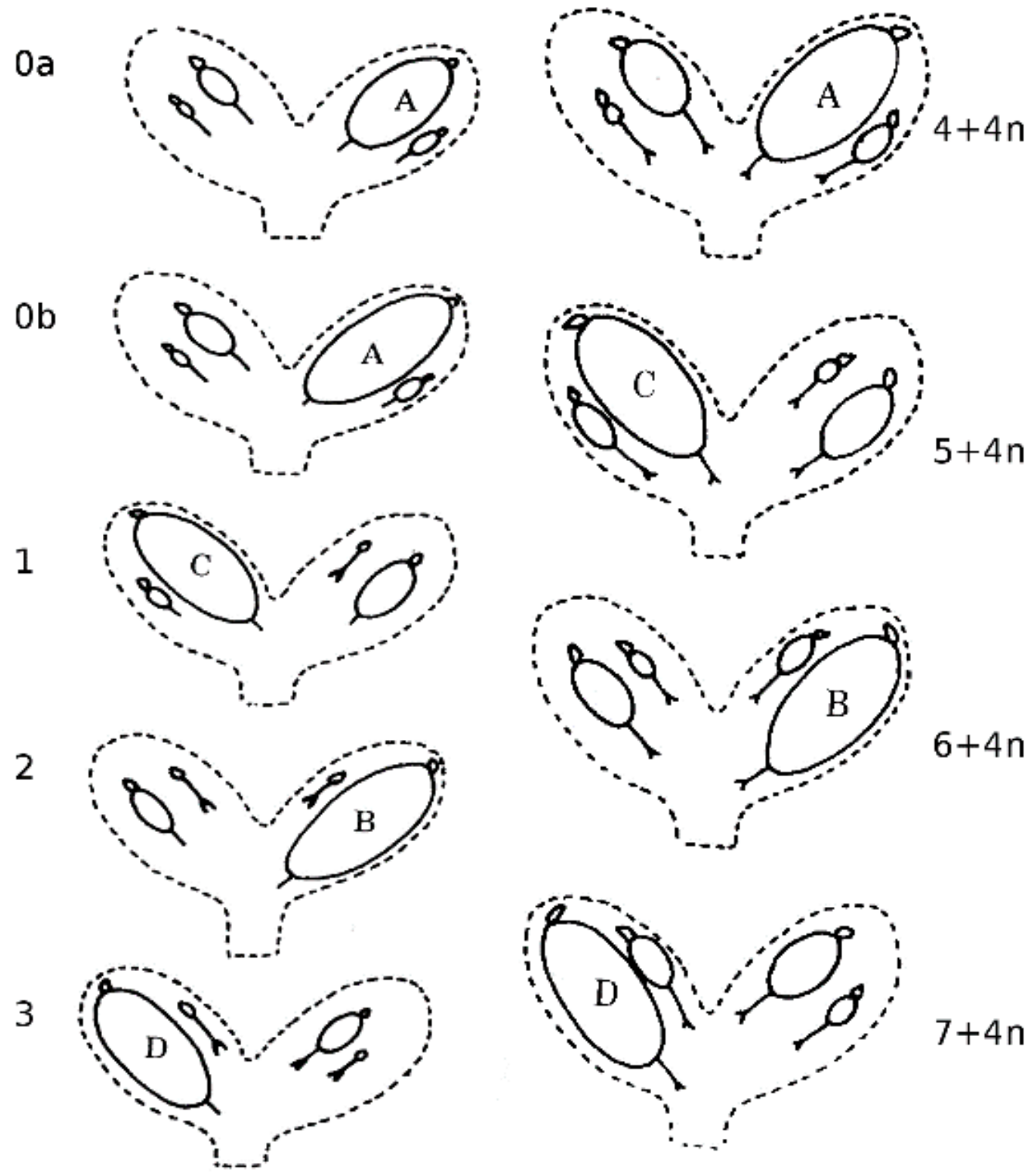

Fig A. Cyclical changes in the oocyte sizes during successive ovulations. Symbols next to each diagram in the left column indicate the number of ovulations completed; those next to the right column indicate the number of ovulations, with the proviso that the variable n can, in principle, take any integer value ≥ 0. Where the "tail" on the end of an oocyte has a "fork" this indicates the presence of follicular relic, indicating that the ovariole has ovulated at least once. If there is no fork, the ovariole has not yet ovulated for the first time.

The relative sizes of the oocytes allows us to distinguish precisely between flies that have ovulated 0, 1, 2 or 3 times. During the first, and each successive, ovarian cycle the four oocytes develop and are ovulated in the order right inner, left inner, right outer, left outer. At the start of the second ovarian cycle, when the female has ovulated 4 times, the four oocytes have the same positions and relative sizes as a fly in ovarian category 0 (**Fig A**). Category 0 and category 4 can, however, be distinguished because ovulation results in the ovariole bearing a follicular relic of the previous ovulation. A fly that has ovulated 4 times has a relic on each ovariole, while a fly in ovarian category zero has no relic on any ovariole. Similarly, flies in ovarian category 1 can be distinguished from flies in ovarian category 5, 2 from 6 and 3 from 7.

Since, however, only one relic is ever seen on an ovariole, further ovulations do not result in the changes in the appearance of the relics and it is thus only possible for flies in ovarian categories 4, 5, 6 and 7 to estimate the number of ovulations modulo 4. Thus, a fly in ovarian category 4 can have ovulated 4, 8, 12, 16 … etc times and similar statements apply to flies in ovarian categories 5, 6 and 7. Accordingly, these flies are designated as belonging to ovarian categories 4+4*n*, 5+4*n*, 6+4*n* and 7+4*n*, respectively.

References

Challier, A. (1965) Amélioration de la méthode de détermination de l'âge physiologique des glossines. Études faites sur *Glossina palpalis palpalis* Vanderplank, 1949. *Bulletin de la Société de Pathologie Exotique*, **58**, 250-259.

Fig B. Steps required to generate life history of female tsetse. Together with means of meteorological variables, during oogenesis, *in utero* development, pupal period and sequential ovulation periods. See **Fig C** for simplified timelines

Date	tbar	tmax	avrh	minrh	avsdmB	maxsdmB	ndvi	rain	Rate	Progress		
17 July 1993	20.92	26.13	58.45	45.70	11.36	18.01	0.3456	0.0	0.08856	3.580	c+pprc	
16 July 1993	21.30	26.11	55.27	42.84	11.97	19.34	0.3483	0.0	0.09054	3.491		
15 July 1993	19.91	26.42	58.16	38.38	10.69	20.66	0.3510	0.0	0.08334	3.401		
14 July 1993	21.38	26.85	56.91	41.50	11.71	20.37	0.3547	0.0	0.09097	3.318		
13 July 1993	23.37	28.71	44.82	30.40	16.66	27.36	0.3583	0.0	0.10133	3.227		
12 July 1993	22.39	29.42	50.23	33.00	14.92	27.37	0.3620	0.0	0.09624	3.125		
11 July 1993	23.27	29.15	49.79	34.94	15.38	26.17	0.3657	0.0	0.10079	3.029		
10 July 1993	22.40	29.61	50.61	33.24	14.76	27.49	0.3693	0.0	0.09630	2.928		
09 July 1993	21.41	28.51	50.12	32.83	14.07	26.03	0.3730	0.0	0.09115	2.832		
08 July 1993	20.77	26.84	57.94	40.75	11.21	20.93	0.3767	0.0	0.08779	2.741		
07 July 1993	20.89	25.53	59.54	43.04	10.51	18.49	0.3803	0.0	0.08843	2.653		
06 July 1993	19.34	25.26	62.38	42.58	9.20	18.45	0.3840	0.0	0.08037	2.565		
05 July 1993	18.49	24.80	67.57	43.81	7.75	17.59	0.3877	0.0	0.07595	2.484		
04 July 1993	19.33	25.42	64.46	44.28	8.70	18.10	0.3913	0.0	0.08029	2.408		
03 July 1993	18.78	22.87	63.66	50.28	8.19	13.86	0.3950	0.0	0.07743	2.328	Rate of pregnancy progression	
02 July 1993	18.74	24.46	62.60	41.42	8.93	17.97	0.3987	0.0	0.07727	2.251	r1 = a1+ b1*(Tbar-T1)	
01 July 1993	20.91	25.67	56.18	40.42	11.35	19.50	0.4023	0.0	0.08855	2.173	a1	0.1046
30 June 1993	20.62	26.60	58.68	36.37	11.43	22.03	0.4060	0.0	0.08704	2.085	b1	0.0052
29 June 1993	20.46	26.49	60.49	43.90	10.32	19.41	0.4097	0.0	0.08619	1.998	T1	24
28 June 1993	18.23	24.52	69.60	46.48	7.29	16.43	0.4133	0.0	0.07460	1.911		
27 June 1993	18.73	23.39	70.61	53.36	6.89	13.41	0.4170	0.0	0.07719	1.837		
26 June 1993	20.20	24.92	61.79	41.15	9.68	18.55	0.4207	0.0	0.08482	1.760		
25 June 1993	20.19	24.47	55.18	42.79	11.21	17.56	0.4243	0.0	0.08481	1.675		
24 June 1993	19.69	25.89	56.74	40.92	10.64	19.68	0.4280	0.0	0.08217	1.590		
23 June 1993	19.40	25.41	52.56	33.64	11.55	21.54	0.4317	0.0	0.08070	1.508		
22 June 1993	19.41	25.76	49.56	38.45	12.09	20.38	0.4353	0.0	0.08075	1.427		
21 June 1993	20.70	27.00	46.91	25.29	14.25	26.57	0.4390	0.0	0.08746	1.346		
20 June 1993	21.95	28.15	53.97	38.87	13.05	23.31	0.4427	0.0	0.09395	1.259		
19 June 1993	21.87	28.46	52.00	35.00	13.59	25.19	0.4463	0.0	0.09350	1.165		
18 June 1993	22.19	28.91	49.19	31.59	14.87	27.26	0.4500	0.0	0.09518	1.072	First day of first pregnancy	
17 June 1993	22.01	28.42	53.09	36.70	13.52	24.36	0.4537	0.0	0.14280	0.976	Day of first ovulation	
16 June 1993	21.19	27.89	57.88	40.47	11.54	22.05	0.4573	0.0	0.14280	0.834		
15 June 1993	20.85	26.77	55.59	39.77	11.84	21.04	0.4610	0.0	0.14280	0.691	Rate of passage	
14 June 1993	19.93	27.04	48.87	31.19	13.34	24.59	0.4640	0.0	0.14280	0.548	to first ovulation	
13 June 1993	21.11	27.62	48.75	32.59	13.99	24.92	0.4671	0.0	0.14280	0.405	r0	0.1428
12 June 1993	20.42	27.59	55.07	35.07	12.03	23.96	0.4701	0.0	0.14280	0.262		
11 June 1993	19.80	26.22	58.29	38.43	10.51	20.97	0.4731	0.0	0.14280	0.120	First day of adult life	
10 June 1993	19.06	25.76	56.95	39.26	10.48	20.13	0.4762	0.0	0.01471	1.000	LAST day of pupal life	
09 June 1993	20.33	26.59	51.83	35.73	12.29	22.00	0.4792	0.0	0.01791	0.985		
08 June 1993	20.51	28.11	54.31	35.38	12.23	24.52	0.4822	0.0	0.01838	0.967		
07 June 1993	19.91	27.12	57.84	38.62	11.00	22.04	0.4853	0.0	0.01680	0.949		
06 June 1993	19.33	25.06	62.75	45.75	9.03	17.25	0.4883	0.0	0.01537	0.932		
05 June 1993	23.13	27.15	59.18	45.76	12.04	19.51	0.4913	0.0	0.02598	0.917		
04 June 1993	24.04	30.73	56.07	40.86	14.17	26.16	0.4944	0.0	0.02878	0.891	k2	0.05689
03 June 1993	22.31	29.11	63.41	40.76	11.04	23.88	0.4974	0.0	0.02350	0.862	a2	5.1737
02 June 1993	21.82	27.22	70.15	52.07	8.39	17.31	0.5004	0.0	0.02206	0.839	b2	-0.2162
01 June 1993	22.73	27.94	62.34	50.19	10.88	18.76	0.5035	0.0	0.02478	0.817		
31 May 1993	24.06	29.00	57.05	42.24	13.38	23.14	0.5065	0.0	0.02884	0.792		
30 May 1993	25.31	31.31	53.97	38.71	15.77	28.03	0.5095	0.0	0.03264	0.763		
29 May 1993	26.34	32.84	47.22	32.11	19.25	33.79	0.5125	0.0	0.03569	0.730		
28 May 1993	26.47	34.16	45.97	28.14	20.41	38.57	0.5156	0.0	0.03608	0.695		
27 May 1993	26.79	34.21	47.50	29.06	20.19	37.82	0.5186	0.0	0.03696	0.658		
26 May 1993	25.71	33.07	53.93	33.41	16.90	33.63	0.5216	0.0	0.03384	0.622		
25 May 1993	25.91	32.54	57.32	39.07	15.63	29.86	0.5247	0.0	0.03446	0.588		
24 May 1993	25.76	32.20	57.32	40.97	15.28	28.39	0.5277	0.0	0.03400	0.553		
23 May 1993	24.83	31.54	54.00	37.01	15.82	29.18	0.5307	0.0	0.03122	0.519	a	1.2061
22 May 1993	23.21	30.73	55.30	36.55	14.12	27.94	0.5338	0.0	0.02623	0.488	b	0.8800
21 May 1993	22.11	29.20	58.80	37.08	12.14	25.42	0.5368	0.0	0.02291	0.462		
20 May 1993	21.98	28.29	61.13	39.09	11.12	23.41	0.5398	0.0	0.02254	0.439		
19 May 1993	21.55	27.73	59.42	45.41	11.24	20.27	0.5429	0.0	0.02127	0.416		
18 May 1993	21.42	27.65	61.03	38.27	11.15	22.86	0.5459	0.0	0.02090	0.395		
17 May 1993	22.75	28.12	64.81	48.37	10.43	19.65	0.5489	0.0	0.02483	0.374		
16 May 1993	23.57	29.64	63.74	43.95	11.50	23.30	0.5520	0.0	0.02732	0.349		
15 May 1993	23.39	30.04	65.53	42.70	11.11	24.34	0.5550	0.0	0.02679	0.322		
14 May 1993	23.22	28.83	67.65	48.80	10.18	20.21	0.5569	0.0	0.02627	0.295		
13 May 1993	24.13	28.80	66.94	51.05	10.51	19.38	0.5588	0.0	0.02905	0.269		
12 May 1993	25.67	30.86	60.57	47.92	13.59	23.21	0.5607	0.0	0.03372	0.240		
11 May 1993	25.86	33.55	53.01	31.79	17.47	35.13	0.5626	0.0	0.03431	0.206		
10 May 1993	24.36	31.68	56.27	34.22	14.85	30.72	0.5645	0.0	0.02978	0.172		
09 May 1993	23.55	30.16	60.60	40.60	12.59	25.26	0.5664	0.0	0.02727	0.142		
08 May 1993	24.76	31.43	55.43	32.57	15.33	31.04	0.5683	0.0	0.03099	0.115		
07 May 1993	24.92	31.54	60.46	36.29	13.76	29.51	0.5702	0.0	0.03147	0.084		
06 May 1993	25.41	31.46	60.36	37.90	13.83	28.64	0.5721	0.0	0.03296	0.052		
05 May 1993	25.21	31.56	62.94	42.86	13.04	26.50	0.5740	0.0	0.03234	0.019	First day of pupal life	
04 May 1993	24.13	30.49	67.08	47.66	10.94	22.84	0.5759	0.0	0.10528	1.000		
03 May 1993	22.90	28.86	70.74	48.37	9.11	20.52	0.5778	0.0	0.09889	0.895		
02 May 1993	22.12	27.46	76.66	50.51	7.15	17.99	0.5797	0.0	0.09482	0.796		
01 May 1993	22.51	27.34	82.06	59.98	5.46	14.56	0.5816	0.0	0.09688	0.701		
30 April 1993	23.63	26.56	80.32	64.72	6.22	11.16	0.5835	31.0	0.10267	0.604		
29 April 1993	27.36	32.63	66.38	52.12	12.95	23.56	0.5854	0.0	0.12210	0.501		
28 April 1993	27.10	33.20	68.66	48.98	12.54	25.95	0.5873	0.0	0.12074	0.379		
27 April 1993	26.17	31.85	72.85	53.66	10.22	21.85	0.5892	0.0	0.11587	0.259		
26 April 1993	25.17	30.35	73.81	57.93	9.09	18.21	0.5911	0.0	0.11071	0.143		
25 April 1993	24.58	29.72	73.94	55.87	8.79	18.43	0.5930	0.0	0.10762	0.032	Start of mother's pregnancy	
24 April 1993	24.38	30.53	71.81	50.18	9.85	21.76	0.5949	0.0	0.10659	1.000		
23 April 1993	24.15	30.10	72.65	50.96	9.36	20.93	0.5968	0.0	0.10537	0.893	Rate of progression of MOTHER'S pregnancy	
22 April 1993	24.10	29.86	74.08	51.91	8.89	20.24	0.5987	0.0	0.10512	0.788	r1 = a1+ b1*(Tbar-T1)	
21 April 1993	24.01	28.00	78.33	61.57	7.08	14.53	0.6006	0.0	0.10463	0.683	a1	0.1046
20 April 1993	25.03	29.68	76.32	58.09	8.14	17.46	0.6025	0.0	0.10995	0.578	b1	0.0052
19 April 1993	25.83	31.31	70.81	53.23	10.71	21.22	0.6044	0.0	0.11410	0.468	T1	24
18 April 1993	25.19	30.91	75.56	55.19	8.79	20.03	0.6063	0.0	0.11079	0.354		
17 April 1993	23.86	28.87	81.32	60.23	6.32	15.81	0.6082	0.0	0.10389	0.243		
16 April 1993	24.21	29.23	83.76	58.58	5.68	16.81	0.6101	0.0	0.10571	0.140		
15 April 1993	22.72	26.95	93.52	75.18	2.14	8.82	0.6120	0.0	0.09792	0.034	Start of growth of oocyte leading to egg	

We start with a fly assumed to have ovulated THREE times, and which has completed 0.58 (58%) of its current pregnancy.

We first "wind back" through this pregnancy, using the formula for the rate of progression of pregnancy - and the mean temperature on each day, to get proportion of pregnancy completed on that day.

We continue this procedure until we get to a value of 1.0 - indicating the time at which the fly has ovulated for the first time.[NOW SCROLL DOWN]

Notice that, at this cool time of the year, pregnancies take about 11 - 12 days

The rate (0.14280) of ovarian development - between adult emergence and first ovulation is approximately independent of temperature - so that the adult female ovulates for the first time at age 7 days.

The rate of pupal development is again temperature dependent, and we wind back to the day of larviposition.

Use these parameter values to take account of the fact that the pupal sites are cooler than indicated by logger temperatures
NB!! Only a2 and b2 are different; k2 is as before

Use following to approximate mean daily temperature in pupal site - as a function of mean daily temperature as measured in a Stevenson screen or logger (Jackson & Phelps, 1967)
T(pup) =a + b*T(logger)

The fly starts life as an oocyte in its mother's ovaries - and then as an egg and developing larva in its mother's uterus.

Note that at the warmer temperatures of April, the pregnancy lasts 10 days - instead of the 12 days of July

Mean values

Period	Start	End	tbar	tmax	avrh	minrh	avsdmB	maxsdmB	ndvi	rain
Adult	11-Jun	17-Jul	20.61	26.58	56.31	38.96	11.65	21.38	0.41	0.00
c=3	11-Jul	17-Jul	21.79	27.54	53.38	38.11	13.24	22.76	0.36	0.00
c=2	30-Jun	10-Jul	20.15	25.96	59.43	40.82	10.56	20.04	0.39	0.00
c=1	18-Jun	29-Jun	20.25	26.11	56.55	39.29	11.29	20.77	0.43	0.00
c=0	11-Jun	17-Jun	20.76	27.36	53.93	36.32	12.39	23.13	0.46	0.00
Pupa	05-May	10-Jun	23.55	29.92	58.46	40.01	13.30	25.70	0.53	0.00
Pregnancy	25-Apr	04-May	24.57	29.85	73.25	53.98	9.25	19.51	0.58	3.10
Oocyte	15-Apr	24-Apr	24.35	29.54	77.82	57.51	7.69	17.76	0.60	0.00

NB!!!! The programme should capture, *for each fly analysed*, all of the inputs and mean values listed in this table

Fig C. Simplified graphic showing the endpoint dates for each of the developmental phases in the life of the example fly.

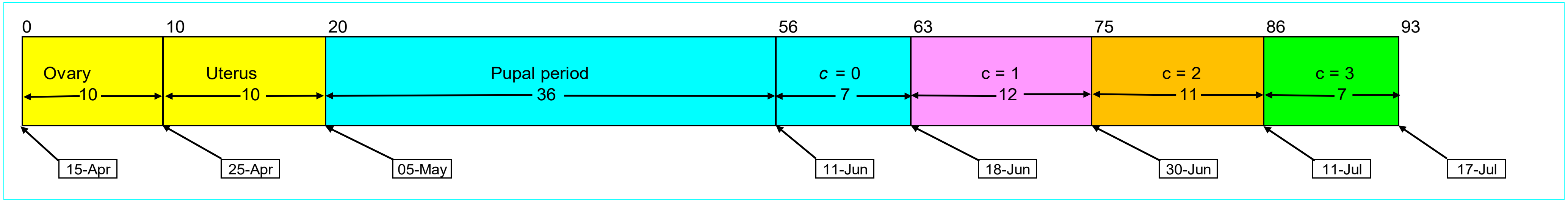

Circannual variation in meteorological variables

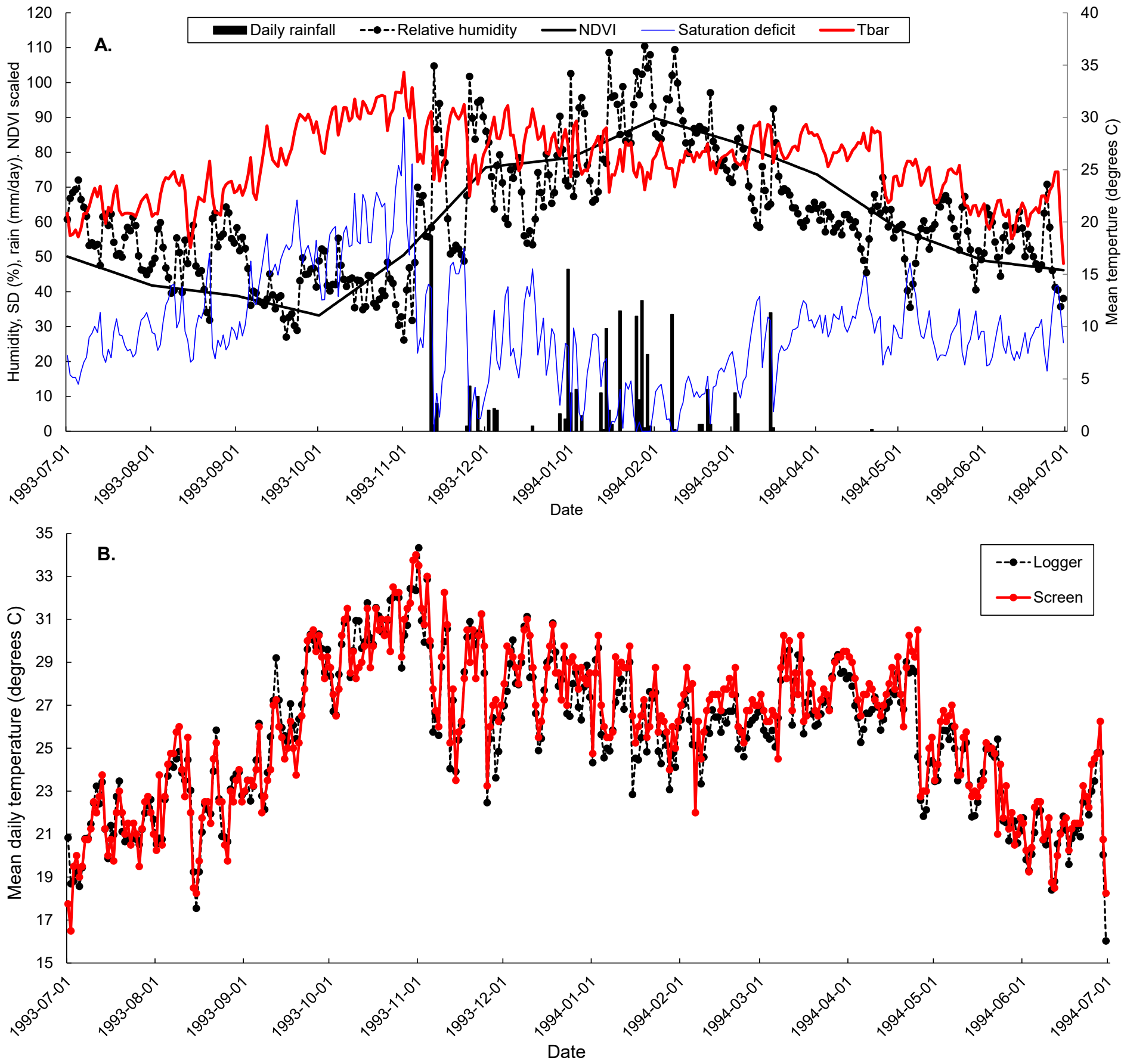

Fig D. Circannual variation in meteorological variables measured at Rekomitjie Research Station, Zambezi Valley, Zimbabwe, for a selected year (1993-94). A. Daily precipitation, average temperature, relative humidity, saturation deficit and NDVI. B. Mean daily temperatures as measured in the Stevenson screen and by the automatic data logger.

Hours per day suitable for tsetse feeding activity

As detailed in the Methods section of the main paper, any hour of a given day was judged suitable for tsetse feeding activity if it lay between the recorded times of sunrise and sunset at Rekomitjie Research Station, and if the mean temperature for that hour was ≥16 and ≤32°C.

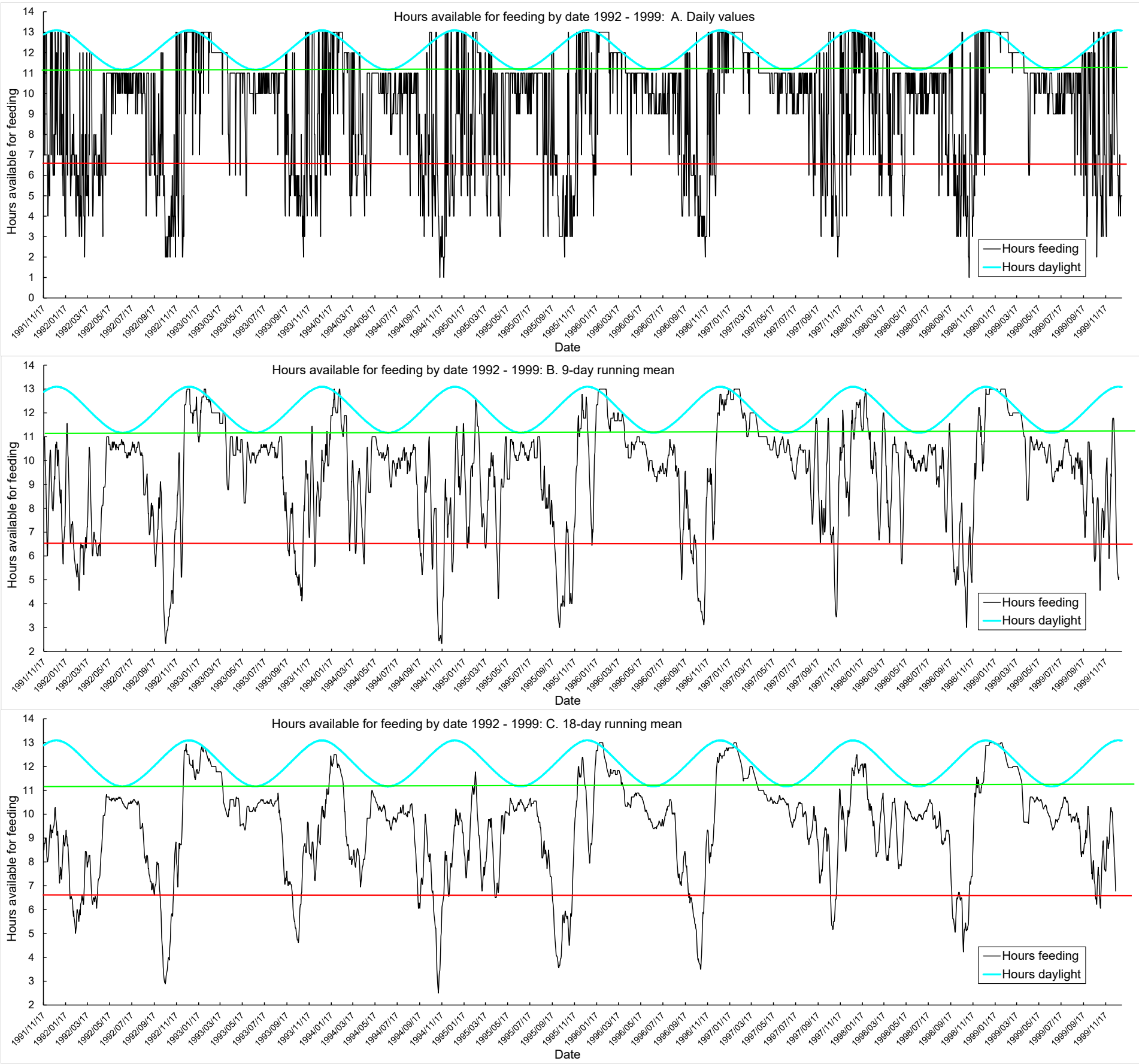

Fig E. Estimated number of hours during each day that are estimated to be suitable for tsetse feeding activity. A. Results for individual days between 17 November 1991 and 31 December 1999. B. 9-day and C. 18-day running means of the daily results. Lines: Blue – Hours of daylight by day. Green – Highest mean monthly hours of feeding (11.1 hours). Red – Lowest mean monthly hours of feeding (6.6 hours).

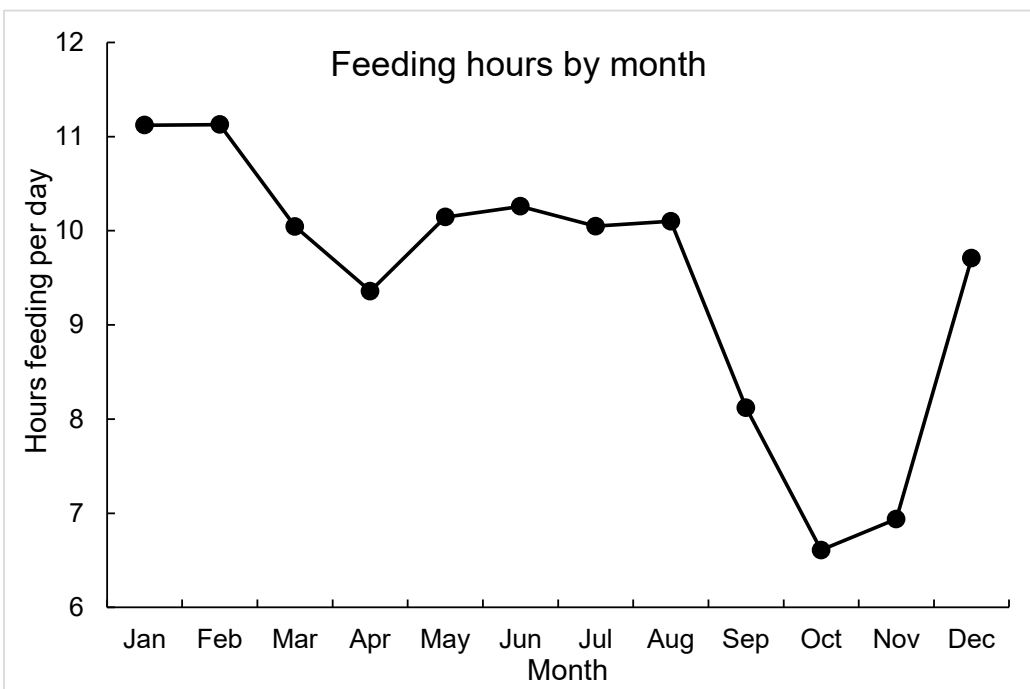

Fig F. Hours suitable for tsetse feeding activity averaged over each month, pooled on years between 1991 and 1999.

Distribution of egg and wing lengths

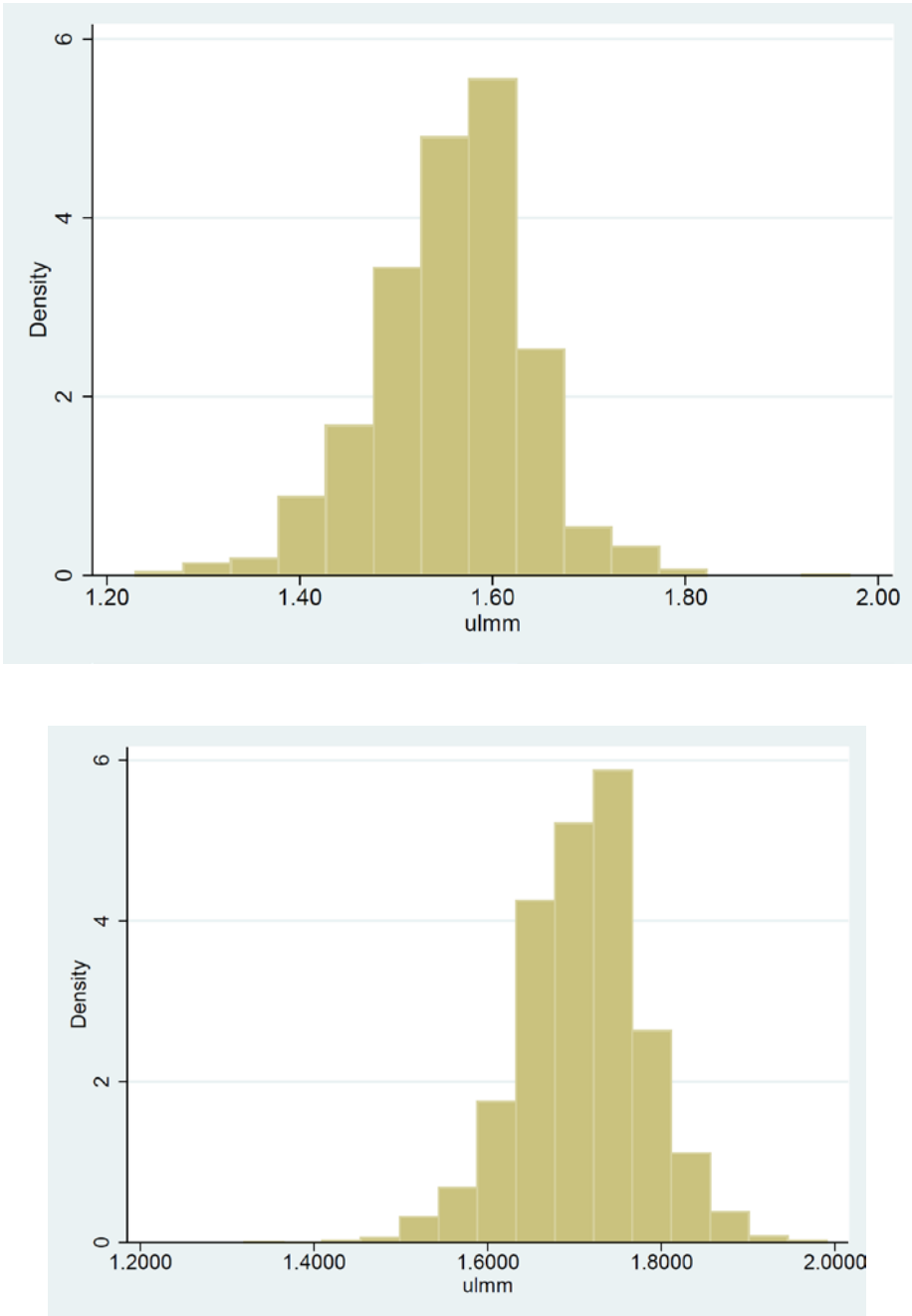

Fig G. Distribution of egg lengths (mm) for *G. m. morsitans* (top) and *G. pallidipes* (bottom) sampled at Rekomitjie Research Station; November 1991 - December 1999.

**Species**	**Observations**	**Mean**	**Std. Err.**	**95% Conf. Interval**
G. pallidipes	40,749	1.7101	0.0004	1.7095 - 1.7108
G. m. morsitans	2,710	1.5550	0.0015	1.5520 - 1.5580

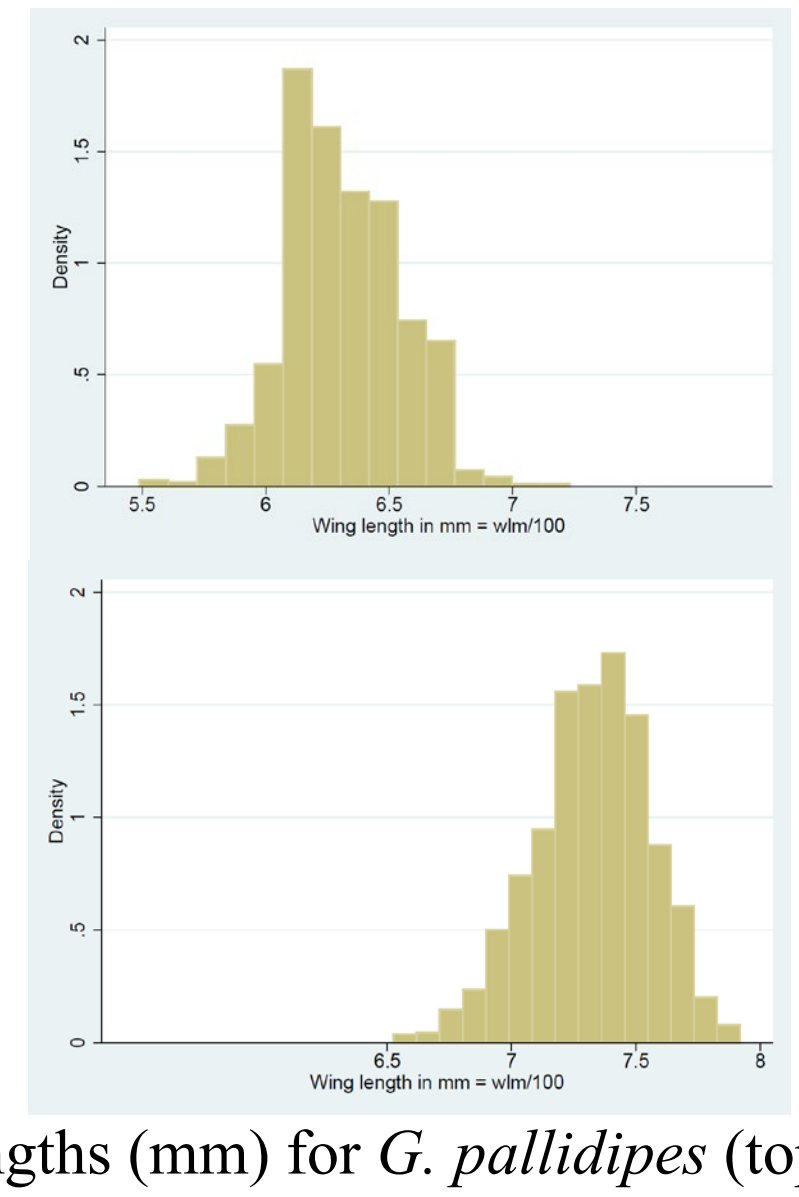

Fig H. Distribution of wing lengths (mm) for *G. pallidipes* (top) and *G. m. morsitans* (bottom) sampled at Rekomitjie Research Station; November 1991 - December 1999. Restricted to flies where the ovarian category $c<4$.

**Species**	**Observations**	**Mean**	**Std. Err.**	**95% Conf. Interval**
G. pallidipes	48,795	7.330	0.001	7.327 7.332
G. m. morsitans	4,520	6.303	0.003	6.296 6.309

Table A. Proportions of flies excluded from analyses because egg or wing lengths were outside reasonable limits for tsetse species in question. Note that all available wing and egg lengths were used for these analyses, so that the sample sizes are larger than seen in Table 3, rows 1 and 7, respectively.

	**Egg length**			
	G. pallidipes		*G. m. morsitans*	
	Bound	# out of bounds	Bound	# out of bounds
Bound				
Upper	200	22	200	4
Lower	130	35	120	2
Total out		57		6
Total in		67,615		6359
GT		67,672		6365
	% out of bounds	0.084%		0.094%

	**Wing length**			
	G. pallidipes		*G. m. morsitans*	
	Bound	# out of bounds	Bound	# out of bounds
Bound				
Upper	800	271	740	0
Lower	650	756	540	4
Total Out		1027		4
Total in		151,508		19,572
GT		152,535		19,576
	% out of bounds	0.67%		0.02%

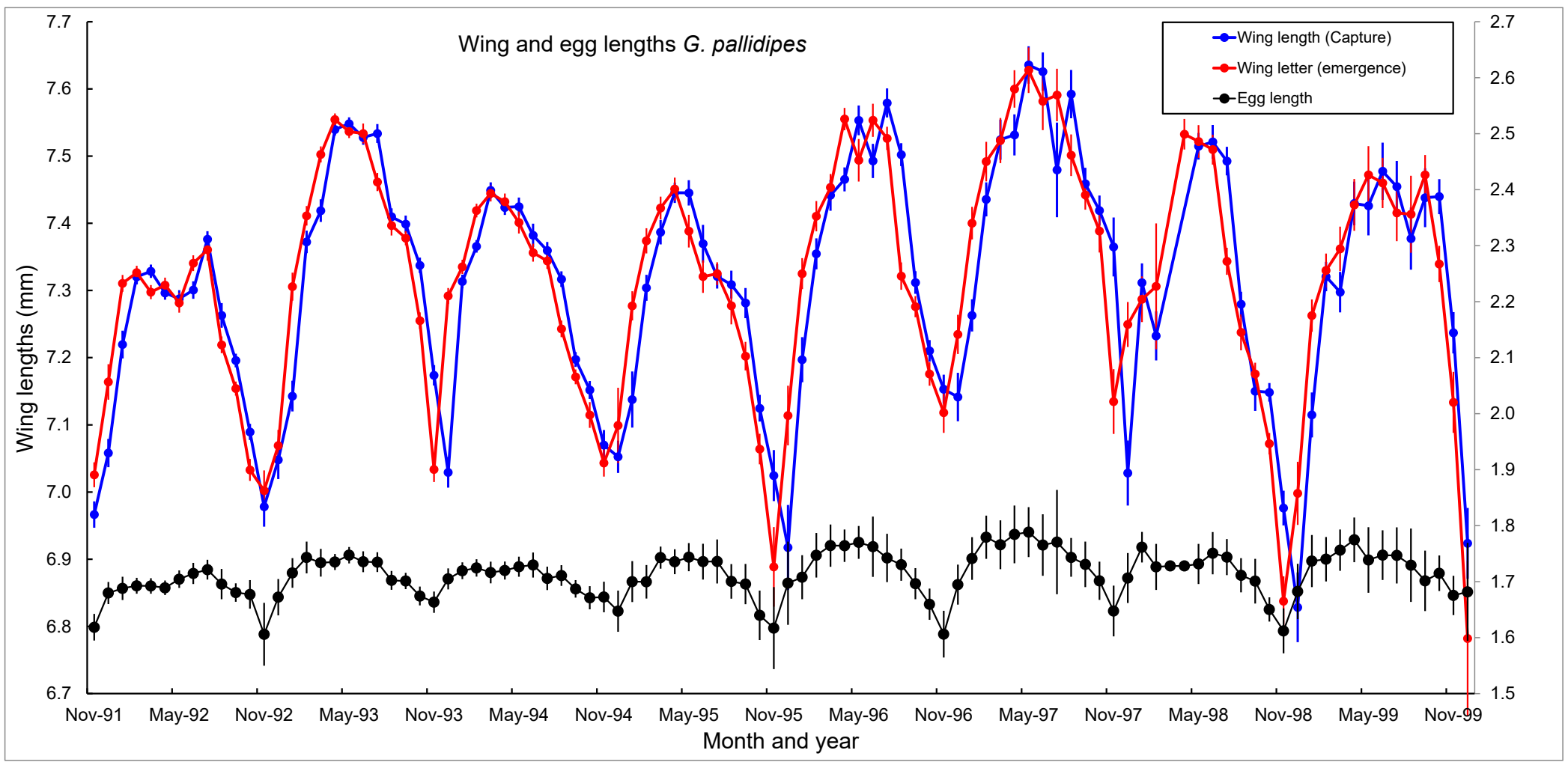

Fig I. Monthly mean wing and egg lengths among female *G. pallidipes* that had ovulated fewer than four times. For wing lengths, flies were pooled over the month when they were captured, or the month when they were estimated to have emerged as adults. Flies sampled in the Zambezi Valley of Zimbabwe; Nov 1991 - Dec 1999.

Egg length vs maternal age and month of capture: monthly pooling

Mean egg lengths for *G. pallidipes* increased sharply with maternal age, in every month, but only between ovarian categories 1 and 3. Thereafter there was little change in egg length with ovarian category (**Fig J** a, b). Egg lengths in every ovarian category decreased consistently between June and November (**Fig J** a). This trend reversed sharply in December and January; thereafter the length was maintained at a relatively constant high maximum level in February – May (**Fig J** b). The patterns were broadly similar for *G. m. morsitans*, though the trends are less obvious given the increased estimation uncertainties, due to the order of magnitude smaller sample size for *G. m. morsitans* (**Fig J** c, d).

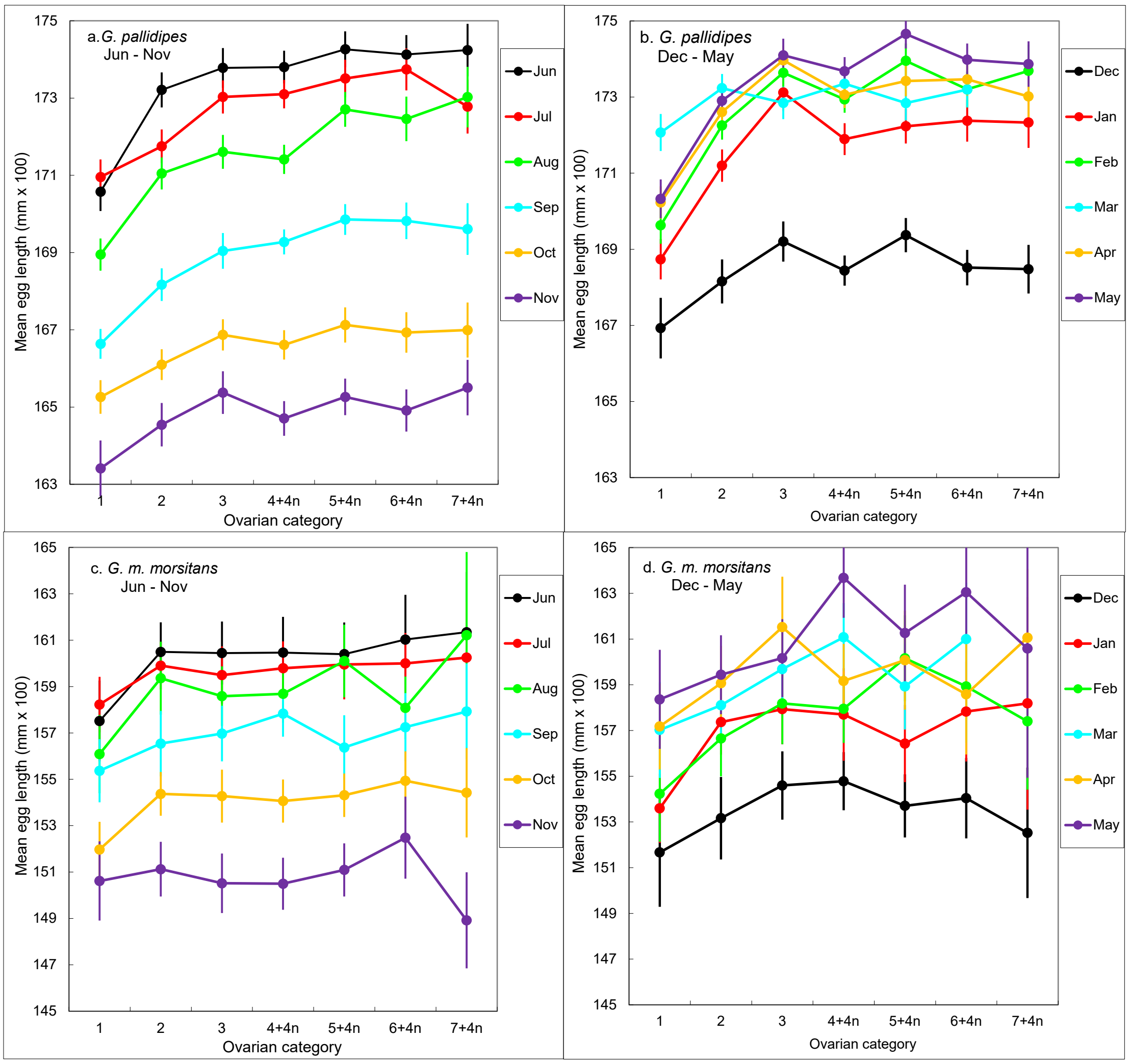

Fig J. Lengths of *in utero* eggs as a function of ovarian category and month of capture, for *G. pallidipes* (n = 64,474) and *G. m. morsitans* (n = 5771) sampled at Rekomitjie Research Station, 1988 – 1999. Sample sizes are smaller than in Table 3, row 7 because, here, we stipulate that, for *G. pallidipes*, individual egg lengths must be in the interval (1.30 – 2.00) mm and for *G. m. morsitans* in the interval (1.20 – 2.00) mm.

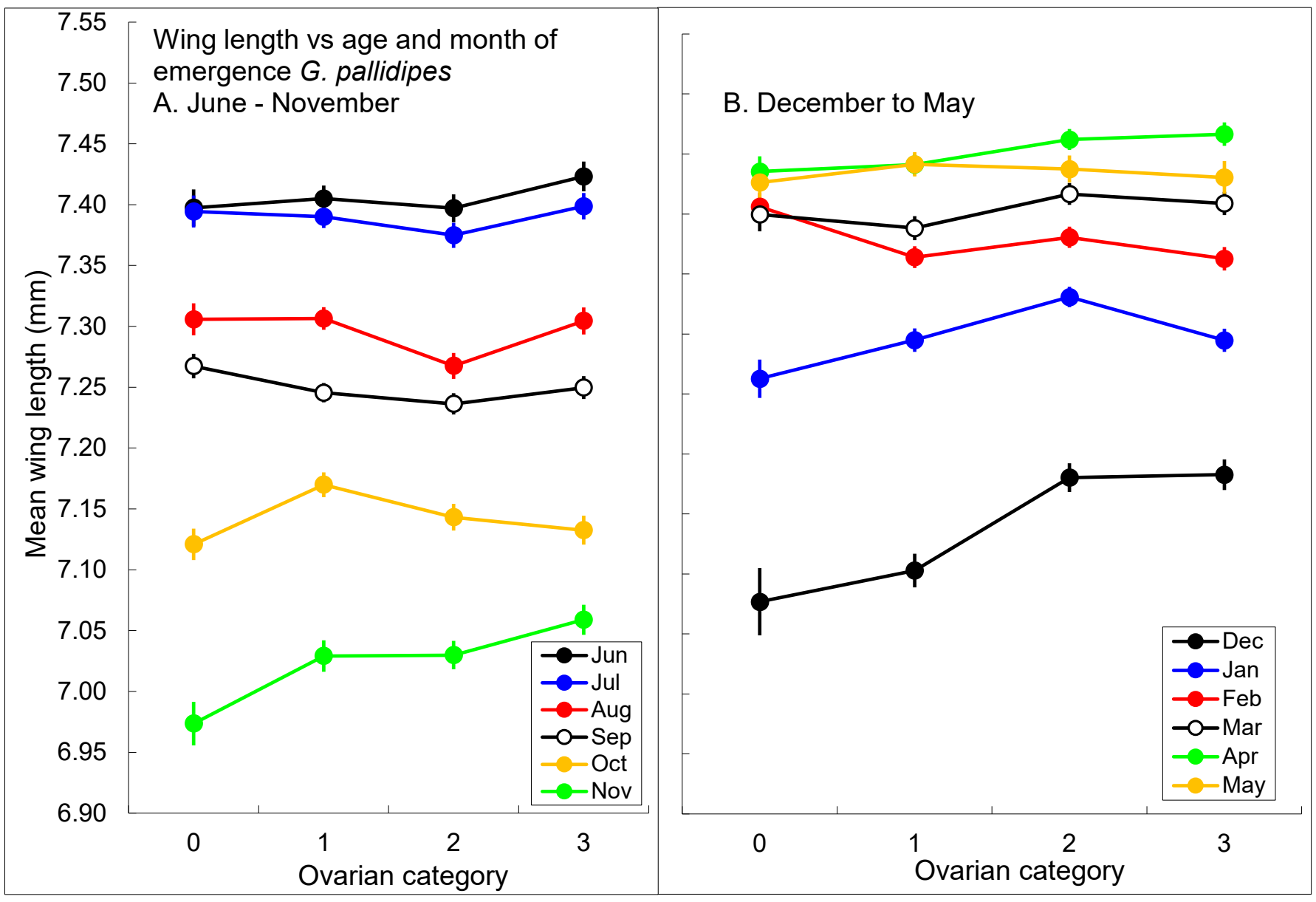

Fig K. Wing length in female *G. pallidipes* sampled at Rekomitjie between Aug 1988 and December 1999, as a function of the age of the fly (ovarian category) and month in which the fly was estimated to have emerged as a teneral adult. Total sample size; 76,421. The sample size is smaller than in Table 3, row 4 because, here, we stipulate that individual wing lengths must be in the interval (5.40 – 8.00) mm.

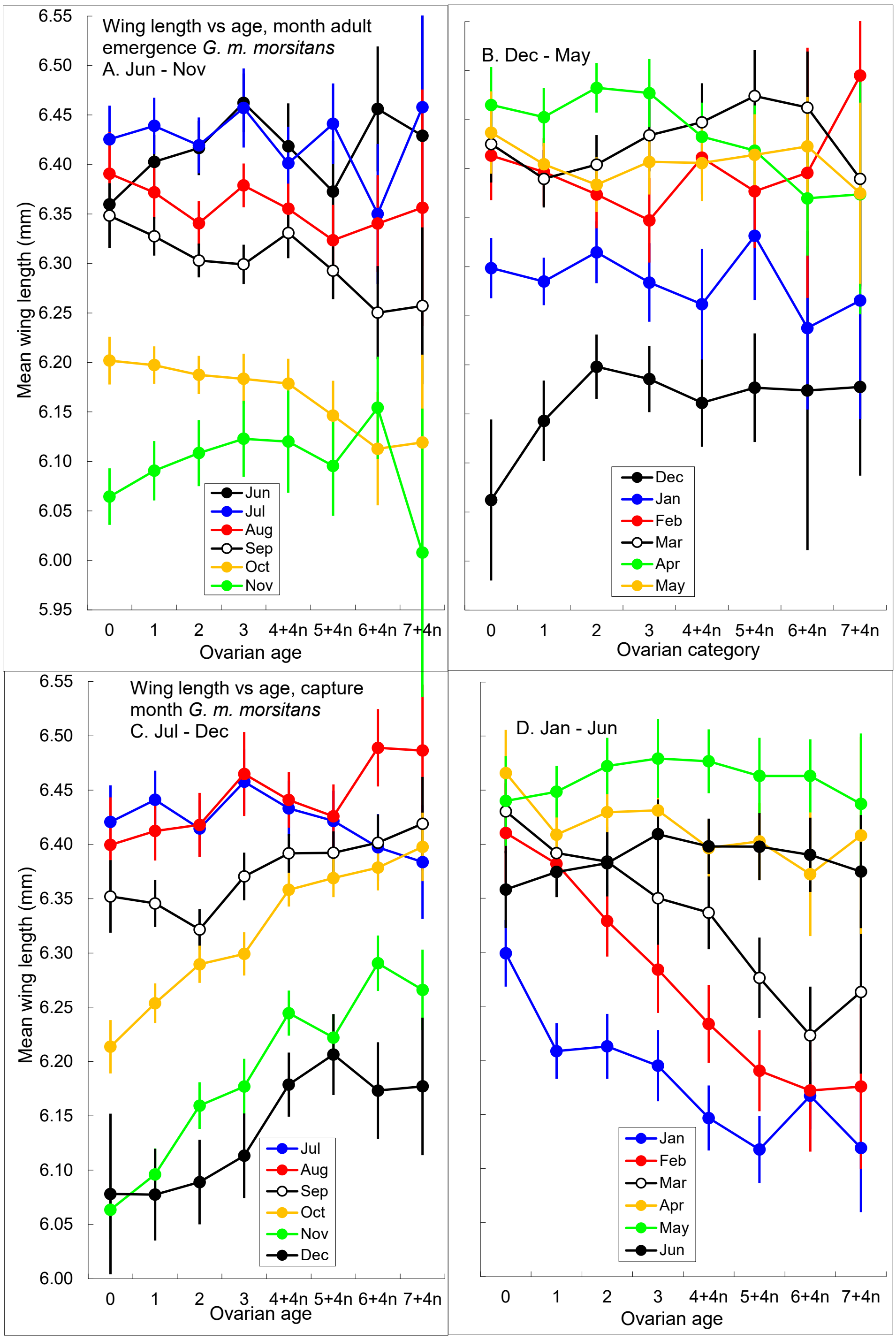

Fig L. Wing length in female *G. m. morsitans* sampled at Rekomitjie between Aug 1988 and December 1999. A, B: As function of the fly's age (ovarian category) and month in which it was estimated to have emerged as a teneral adult. Total sample size; 13,999. C, D: As function of the ovarian age of the fly and month in which the fly was captured. Total sample size; 19, 569, *cf* Table 3, row 1. Sample sizes are smaller in C and D because, for $c > 3$, it was not possible to gauge the month of emergence.

Analyses of factors affecting egg lengths in individual *G. pallidipes* and *G. m. morsitans*

Individual flies

We only use data for flies captured on days when the logger was functional, so that values were available for every independent meteorological variable. We also stipulated that the ovarian category was in the range [1-7] – *i.e.*, we excluded flies of unknown ovarian category – and those in ovarian category zero, which did not have an egg *in utero*.

To exclude flies where it seemed likely either that there had been an error in the measurement of egg or wing length, or where the fly had been assigned to the wrong species, we set the following bounds for the measures of the acceptable lengths of the egg (*ulm*) and wing (*wlm*):

G. pallidipes: $130 \le ulm \le 200$ and $650 \le wlm \le 800$

G. m. morsitans: $120 \le ulm \le 200$ and $540 \le wlm \le 740$

This resulted in the exclusion of flies with out of bounds measurements in <0.7% of flies in the case of wing lengths, and <0.1% for egg lengths (**Table A**).

Egg length	*ulmm*	mm
Saturation deficit	*SDbar*	millibars
Hours suitable for feeding	*HrsFeed*	hours
Relative humidity	*RHbar*	percent
Mean temperature	*Tbar*	degrees C
NDVI	*ndvi*	-
Maternal wing length	*wlmm*	mm
Ovarian category	*c*	-
Rain	*rain*	mm/day

Product-moment correlations between egg length (*ulmm*) and eight candidate explanatory variables, for the period when logger data were available. Variables are sorted, left to right, in decreasing correlation magnitude with *ulmm*, as given in the top row. Maternal ovarian category (*ovcat*) is treated as an integer. Each variable of the form [*var*].mn is the average, for each fly, of [*var*] over that fly's estimated time period of egg development.
Entries in the upper-right (unshaded) triangular portion of each matrix are the raw product-moment correlations (*r*); those in the lower-left (yellow-shaded cells) provide the r^2 values.

Table B. *G. pallidipes* *n* = 40,749

	ulmm	*SDbar*	HrsFeed	*RHbar*	*Tbar*	*ndvi*	*wlmm*	*ovcat*	*rain*
ulmm	1.00	-0.35	0.31	0.26	-0.26	0.23	0.21	0.13	0.06
SDbar.mn	-0.35	1.00	-0.83	-0.89	0.47	-0.64	-0.11	0.01	-0.44
HrsFeed.mn	0.31	-0.83	1.00	0.62	-0.60	0.30	0.10	-0.03	0.31
RHbar.mn	0.26	-0.89	0.62	1.00	-0.03	0.78	-0.02	0.02	0.58
Tbar.mn	-0.26	0.47	-0.60	-0.03	1.00	0.16	-0.29	0.05	0.11
ndvi.mn	0.23	-0.64	0.30	0.78	0.16	1.00	0.01	0.02	0.34
wlmm	0.21	-0.11	0.10	-0.02	-0.29	0.01	1.00	-0.03	-0.15
ovcat	0.13	0.01	-0.03	0.02	0.05	0.02	-0.03	1.00	0.02
rain.mn	0.06	-0.44	0.31	0.58	0.11	0.34	-0.15	0.02	1.00

Table C. *G. m. morsitans* *n* = 2710

	ulmm	*Tbar*	*HrsFeed*	*SDbar*	*wlmm*	RHbar	*ndvi*	ovcat	*rain*
ulmm	1.00	-0.41	0.33	-0.29	0.25	0.12	0.10	0.06	-0.02
Tbar.mn	-0.41	1.00	-0.77	0.69	-0.24	-0.27	-0.14	0.05	-0.01
HrsFeed.mn	0.33	-0.77	1.00	-0.92	0.10	0.70	0.46	-0.02	0.35
SDbar.mn	-0.29	0.69	-0.92	1.00	-0.09	-0.87	-0.66	0.03	-0.45
wlmm	0.25	-0.24	0.10	-0.09	1.00	-0.03	0.00	0.01	-0.12
RHbar.mn	0.12	-0.27	0.70	-0.87	-0.03	1.00	0.77	-0.01	0.62
ndvi.mn	0.10	-0.14	0.46	-0.66	0.00	0.77	1.00	-0.05	0.40
ovcat	0.06	0.05	-0.02	0.03	0.01	-0.01	-0.05	1.00	-0.02
rain.mn	-0.02	-0.01	0.35	-0.45	-0.12	0.62	0.40	-0.02	1.00

Key: *ulmm*, Egg length (mm); *SDbar*, saturation deficit (mB); *HrsFeed*, Hours per day suitable for feeding activity; *Tbar*, Mean daily temperature (degrees C); *RHbar*, Mean daily relative humidity (%); *wlmm*, Wing length (mm); *ndvi*, Normalised Difference Vegetation Index; *ovcat*, Ovarian category, with all flies of category 4 or older assigned to category 4.

Full-model regression of egg length (mm) for individual flies. The predictor variables are the same list as in the above correlations. Here, ovarian category, (*ovcat*) is treated as a category, not an integer. In defining *ovcat*, all flies of category 4 or older were assigned to category 4.

Table D. ***G. pallidipes*** (*n* = 40,749).

Call: lm(formula = *ulmm* ~ *wlmm* + *Tbar*.mn + *RHbar*.mn + ovcat + *SDbar*.mn + HrsFeed.mn + *ndvi*.mn + *rain*.mn, data = modl.9199.dat)

Residuals:	Min	1Q	Median	3Q	Max
	-0.381	-0.0409	0.00297	0.0431	0.335

Coefficients:	Estimate	Std. Error	t value	Pr(>|t|)
(Intercept)	1.42e+00	1.47e-02	96.9	< 2e-16 ***
wlmm	4.25e-02	1.42e-03	30.0	< 2e-16 ***
Tbar.mn	-3.47e-03	4.22e-04	-8.22	< 2e-16 ***
RHbar.mn	-8.01e-05	1.48e-04	-0.54	0.588
ovcat2	2.00e-02	1.16e-03	17.2	< 2e-16 ***
ovcat3	3.23e-02	1.19e-03	27.2	< 2e-16 ***
ovcat4	3.07e-02	9.51e-04	32.2	< 2e-16 ***
SDbar.mn	-1.00e-03	4.35e-04	-2.30	0.0213 *
HrsFeed.mn	3.52e-03	3.38e-04	10.4	< 2e-16 ***
ndvi.mn	7.59e-02	3.90e-03	19.4	< 2e-16 ***
rain.mn	-5.96e-04	1.34e-04	-4.45	8.79e-06 ***

Residual standard error: 0.0644 on 40738 degrees of freedom

Multiple R^2: 0.187, Adjusted R^2: 0.187 F-statistic: 938.8 on 10 and 40738 DF, p < 2.2e-16

Table E. ***G. m. morsitans*** (*n* = 2710)

Call: lm(formula = *ulmm* ~ *wlmm* + *Tbar*.mn + *RHbar*.mn + ovcat + *SDbar*.mn + *HrsFeed*.mn + *ndvi*.mn + *rain*.mn, data = modl.9199.dat)

Residuals:	Min	1Q	Median	3Q	Max
	-0.306	-0.0434	-0.00169	0.04394	0.37279

Coefficients:	Estimate	Std. Error	t value	Pr(>|t|)
(Intercept)	1.303	0.063	20.7	< 2e-16 ***
wlmm	0.0573	0.00616	9.31	< 2e-16 ***
Tbar.mn	-0.00855	0.00190	-4.51	6.85e-06 ***
RHbar.mn	0.000107	0.000644	0.166	0.868
ovcat2	0.0157	0.00495	3.17	0.00153 **
ovcat3	0.0237	0.00513	4.62	4.09e-06 ***
ovcat4	0.0213	0.00412	5.16	2.68e-07 ***
SDbar.mn	0.00187	0.00192	0.970	0.332
HrsFeed.mn	0.00535	0.00188	2.85	0.00444 **
ndvi.mn	0.0451	0.0164	2.76	0.00590 **
rain.mn	-0.000594	0.000718	-0.828	0.408

Residual standard error: 0.0704 on 2699 degrees of freedom

Multiple R^2: 0.209, Adjusted R^2: 0.206 F-statistic: 71.4 on 10 and 2699 DF, p-value: < 2.2e-16

Regressions on pooled data

Regression models, applying all candidate predictors to pooled data.

Table F. *G. pallidipes:* Weekly pooled data

Call: lm(formula = *ulmm*.wkmn ~ *wlmm*.wkmn + *Tbar*.wkmn + *RHbar*.wkmn + *SDbar*.wkmn + *ndvi*.wkmn + *rain*.wkmn + *HrsFeed*.wkmn, data = modl.dat)

Residuals:	Min	1Q	Median	3Q	Max
	-0.0505	-0.0128	-0.00213	0.0116	0.0903

**Coefficients**	**Estimate**	**Std. Error**	**t value**	**Pr(>|t|)**
(Intercept)	1.016	0.0869	11.7	< 2e-16 ***
wlmm.wkmn	0.0935	0.00975	9.59	< 2e-16 ***
Tbar.wkmn	-0.00261	0.00139	-1.88	0.0612
RHbar.wkmn	0.000227	0.000481	0.473	0.637
SDbar.wkmn	-0.000571	0.00148	-0.386	0.699
ndvi.wkmn	0.0689	0.0133	5.200	3.48e-07 ***
rain.wkmn	-0.000573	0.000455	-1.259	0.209
HrsFeed.wkmn	0.00508	0.00141	3.598	0.00037 ***

Residual standard error: 0.0203 on 333 degrees of freedom

Multiple R^2: 0.774, Adjusted R^2: 0.769 F-statistic: 162.8 on 7 and 333 DF, *p*: < 2.2e-16

Table G. *G. m. morsitans*: Monthly pooled data.

Call: lm(formula = *ulmm*.momn ~ *wlmm*.momn + *Tbar*.momn + *RHbar*.momn + *SDbar*.momn + *ndvi*.momn + *rain*.momn + *HrsFeed*.momn, data = modl.dat)

Residuals:	Min	1Q	Median	3Q	Max
	-0.0587	-0.0139	-0.00278	0.0148	0.0756

**Coefficients**	**Estimate**	**Std. Error**	**t value**	**Pr(>|t|)**
(Intercept)	0.763	0.265	2.88	0.00567 **
wlmm.momn	0.125	0.0333	3.76	0.000406 ***
Tbar.momn	-0.00945	0.00536	-1.77	0.083 .
RHbar.momn	0.00203	0.00187	1.08	0.283
SDbar.momn	0.00500	0.00538	0.929	0.357
ndvi.momn	0.0197	0.0426	0.461	0.647
rain.momn	-0.00232	0.00189	-1.22	0.226
HrsFeed.momn	0.00651	0.00593	1.10	0.277

Residual standard error: 0.0276 on 57 degrees of freedom

Multiple R^2: 0.656, Adjusted R^2: 0.614

F-statistic: 15.5 on 7 and 57 DF, p-value: 3.40e-11

Analyses of factors affecting wing lengths in female *G. pallidipes*

Wing length regressions for young flies (*c*<4) only.

Table H. Regression of wing length on 6 environmental predictors, for the period (1992 – 1999), for individual flies (*n* = 48,795).

Call: lm(formula = Length ~ *Tbar*.mn + *RHbar*.mn + *SDbar*.mn + *ndvi*.mn + *rain*.mn + *HrsFeed*.mn, data = perfly.log)

Residuals:

Min	1Q	Median	3Q	Max
-1.44632	-0.13312	0.00847	0.14243	1.31489

Coefficients :

	Estimate	Std. Error	t value	Pr(>|t|)
(Intercept)	7.437e+00	3.399e-02	218.758	< 2e-16 ***
Tbar.mn	1.033e-03	1.499e-03	0.689	0.49109
RHbar.mn	-5.754e-05	5.402e-04	-0.107	0.91518
SDbar.mn	-1.631e-02	1.598e-03	-10.201	< 2e-16 ***
ndvi.mn	3.464e-01	1.267e-02	27.345	< 2e-16 ***
rain.mn	-5.912e-03	4.176e-04	-14.158	< 2e-16 ***
HrsFeed.mn	-3.631e-03	1.387e-03	-2.618	0.00884 **

Residual standard error: 0.2179 on 48,268 degrees of freedom

520 observations deleted (missing values of *wlmm*)

Multiple R-squared: 0.2356, Adjusted R-squared: 0.2355

F-statistic: 2480 on 6 and 48268 DF, p-value: < 2.2e-16

Table I. Correlation matrix of *G. pallidipes* wing length versus 6 environmental variables for the period (1992 – 1999), for individual flies (n = 48,795). Variables are sorted left to right, in descending order of correlation strength with *Length*.

	Length	*SDbar*.mn	*RHbar*.mn	*ndvi*.mn	*HrsFeed*.mn	*rain*.mn	*Tbar*.mn
Length	1.00	-0.45	0.43	0.42	0.33	0.19	-0.07
SDbar.mn	-0.45	1.00	-0.87	-0.62	-0.86	-0.52	0.36
RHbar.mn	0.43	-0.87	1.00	0.78	0.62	0.67	0.12
ndvi.mn	0.42	-0.62	0.78	1.00	0.29	0.41	0.27
HrsFeed.mn	0.33	-0.86	0.62	0.29	1.00	0.37	-0.54
rain.mn	0.19	0.52	0.67	0.41	0.37	1.00	0.19
Tbar.mn	-0.07	0.36	0.12	0.27	-0.54	0.19	1.00

Weekly pooling

G. pallidipes -- Data for all regressions are weekly means for the 414 weeks when logger data are available, 1991-99.

Table J. Matrix of correlations between mean wing length and 6 predictor variables. Columns are sorted in decreasing order of correlation strength with *length*, as shown in the first row.

	Length	*SDbar*	*RHbar*	*ndvi*	*HrsFeed*	*Tbar*	*rain*
Length	1.00	-0.80	0.73	0.69	0.67	-0.38	0.29
SDbar	-0.80	1.00	-0.88	-0.69	-0.91	0.54	-0.47
RHbar	0.73	-0.88	1.00	0.81	0.73	-0.09	0.67
ndvi	0.69	-0.69	0.81	1.00	0.51	0.00	0.40
HrsFeed	0.67	-0.91	0.73	0.51	1.00	-0.60	0.41
Tbar	-0.38	0.54	-0.09	0.00	-0.60	1.00	0.17
rain	0.29	-0.47	0.60	0.40	0.41	0.17	1.00

Saturation deficit (*SDbar.wkmn)* has the strongest correlation with mean wing length. Note the high correlations among various predictor variables.

Table K. Full regression on all 6 predictors.

Call: lm(formula = length.wkmn ~ *Tbar*.wkmn + *RHbar*.wkmn + <u>*SDbar*</u>.wkmn + *ndvi*.wkmn + *rain*.wkmn + *HrsFeed*.wkmn, data = wkmn.log)

Residuals:	Min	1Q	Median	3Q	Max
	-0.322	-0.0587	0.00350	0.0630	0.243

Coefficients:	Estimate	Std. Error	t value	Pr(>|t|)
(Intercept)	7.71e+00	1.54e-01	50.19	< 2e-16***
Tbar.wkmn	4.62e-04	5.82e-03	0.08	0.94
RHbar.wkmn	-1.79e-05	2.16e-03	-0.01	0.994
SDbar.wkmn	-2.24e-02	6.31e-03	-3.55	0.0004***
ndvi.wkmn	2.60e-01	5.81e-02	4.47	9.96e-06***
rain.wkmn	-8.17e-03	2.42e-03	-3.38	0.0008***
HrsFeed.wkmn	-1.76e-02	6.79e-03	-2.60	0.0098**

Residual standard error: 0.097 on 407 degrees of freedom

Multiple R-squared: 0.71, Adjusted R-squared: 0.7008

F-statistic: 162.3 on 6 and 407 DF, p-value: < 2.2e-16 AIC = -1922.2

Analyses of factors affecting wing lengths in female *G. m. morsitans*

Analysis of data for individual flies

G. m. morsitans

Table L. Regression of *G. m. morsitans* wing length on 6 environmental predictors, for the period (1992 – 1999), for individual flies (n = 4520). Using only flies where we have a life history and where *c*<4

Call: lm(formula = *wlmm* ~ *Tbar*.mn + *RHbar*.mn + *SDbar*.mn + *ndvi*.mn + *rain*.mn + *HrsFeed*.mn, data = perfly.log)

Residuals:

Min	1Q	Median	3Q	Max
-1.02539	-0.13528	-0.00569	0.13973	0.99633

Coefficients :

	Estimate	Std. Error	t value	Pr(>|t|)
(Intercept)	6.445e+00	1.023e-01	62.989	< 2e-16 ***
Tbar.mn	-6.366e-03	4.412e-03	-1.443	0.1491
RHbar.mn	-1.015e-03	1.538e-03	-0.660	0.5091
SDbar.mn	-7.864e-03	4.574e-03	-1.719	0.0856 .
ndvi.mn	4.056e-01	4.941e-02	8.207	2.92e-16 ***
rain.mn	-2.582e-05	1.884e-03	-0.014	0.9891
HrsFeed.mn	5.859e-03	4.606e-03	1.272	0.2034

Residual standard error: 0.2123 on 4509 degrees of freedom
4 observations deleted (missing values of *wlmm*)
Multiple R-squared: 0.1876, Adjusted R-squared: 0.1865
F-statistic: 173.5 on 6 and 4509 DF, p-value: < 2.2e-16

Table M. Correlation matrix of *G. m. morsitans* wing length versus 6 environmental variables for the period (1992 – 1999), for individual flies (n = 4520). Variables are sorted left to right, in descending order of correlation strength with Length.

	Length	*SDbar*.mn	*RHbar*.mn	*ndvi*.mn	*HrsFeed*.mn	*Tbar*.mn	*rain*.mn
Length	1.00	-0.41	0.37	0.36	0.35	-0.20	0.17
SDbar.mn	-0.41	1.00	0.86	0.68	-0.88	0.53	-0.47
RHbar.mn	0.37	-0.86	1.00	0.84	0.66	-0.05	0.67
ndvi.mn	0.36	-0.68	0.84	1.00	0.43	0.08	0.41
HrsFeed.mn	0.35	-0.88	0.66	0.43	1.00	-0.60	0.38
Tbar.mn	-0.20	0.53	-0.05	0.08	-0.60	1.00	0.18
rain.mn	0.17	-0.47	0.67	0.41	0.38	0.18	1.00

Analysis of data pooled by month

Data for regressions are monthly means for 87 months when logger data is available, 1991-99.

Table N. Matrix of correlations between mean wing length and 6 predictor variables. Columns are sorted in decreasing order of correlation strength with *length.momn*, as shown in the first row. Using only flies where we have a life history and where *c*<4

	length.momn	*SDbar*.momn	*HrsFeed*.momn	*RHbar*.momn	*ndvi*.momn	*Tbar*.momn	rain.momn
length.momn	1.00	-0.79	0.71	0.67	0.66	-0.48	0.29
SDbar.momn	-0.79	1.00	-0.93	-0.89	-0.78	0.52	-0.53
HrsFeed.momn	0.71	-0.93	1.00	0.76	0.65	-0.56	0.50
RHbar.momn	0.67	-0.89	0.76	1.00	0.86	-0.09	0.74
ndvi.momn	0.66	-0.78	0.65	0.86	1.00	-0.09	0.49
Tbar.momn	-0.48	0.52	-0.56	-0.09	-0.09	1.00	0.20
rain.momn	0.29	-0.53	0.50	0.74	0.49	0.20	1.00

Saturation deficit (*SDbar.momn)* again has the strongest correlation with mean wing length. Note the high correlations among various predictor variables

Table O. Full regression model using all 6 predictors. Using only flies where we have a life history and where *c*<4

lm(formula = length.momn ~ *Tbar*.momn + *RHbar*.momn + *SDbar*.momn + *ndvi*.momn + *rain*.momn + *HrsFeed*.momn, data = momn.log)

Residuals:	Min	1Q	Median	3Q	Max
	-0.268	-0.0516	-0.000021	0.0527	0.186

:

Coefficients	Estimate	Std. Error	t value	Pr(>|t|)
(Intercept)	6.638	0.358	18.5	<2e-16 ***
Tbar.momn	-0.00523	0.0118	-0.442	0.660
RHbar.momn	0.000961	0.00457	0.210	0.834
SDbar.momn	-0.0133	0.0138	-0.962	0.339
ndvi.momn	0.128	0.127	1.01	0.317
rain.momn	-0.00730	0.00596	-1.223	0.225
HrsFeed.momn	-0.00739	0.0157	-0.472	0.638

Residual standard error: 0.0799 on 80 degrees of freedom
Multiple R-squared: 0.663, Adjusted R-squared: 0.637
F-statistic: 26.2 on 6 and 80 DF, p-value: < 2.2e-16 AIC = -433.1

Sample sizes in previous studies

Fig M. Sample sizes, from published studies, of field-caught adult tsetse and teneral adults emerging in the laboratory from field-collected pupae.

Jackson (1948)

			Total recaptures	Totals
G. morsitans	Males	Teneral	63	
		NT	161	196
		**Total**	**224**	
	Female	Teneral	48	
		NT	69	
		**Total**	**117**	
G. swynnertoni	Males	Teneral	199	
		NT	249	423
		**Total**	**448**	
	Female	Teneral	129	
		NT	76	
		**Total**	**205**	

Jackson (1953)

	Year	Jna	Feb	Jan	Mar	Jna	Apr	Jna	May	Jna	Jun	Jna	Jul		Totals
G. pallidipes	**1945**	0	0	0	0	0	0	0	0	0	8	14	8		30
	**1946**	32	11	11	41	55	59	33	12	0	16	4	16		290
[Males only]	**1947**	10	20	50	27	20	7	13	14	15	10	7	4		197
	**1948**	7	5	11	21	11	4	4	3	7	6	8	4		91
														GT	608
G. morsitans	**1946**	0	0	0	0	0	0	0	0	0	0	45	96		141
	**1947**	34	58	86	46	34	42	21	41	50	50	9	28		499
[Males only]	**1948**	44	50	28	12	18	54	28	47	47	50	50	52		480
															0
														GT	1120
G. swynnertoni	**1945**	0	0	0	0	0	0	0	0	15	221	108	89		433
	**1946**	98	102	52	301	201	178	247	89	58	98	68	50		1542
[Males only]	**1947**	50	50	50	50	50	0	37	51	67	50	50	50		555
	**1948**	22	35	30	32	51	39	31	48	88	24	25	41		466
														GT	2996

Bursell & Glasgow (1960)

	Year	Jan	Feb	Mar	Apr	May	Jun	Jul	Aug	Sep	Oct	Nov	Dec		Totals
G. fuscipes	1955	0	0	0	0	0	0	0	0	0	185	0	0		185
	1956	178	0	0	209	222	0	0	0	0	0	0	0		609
[Males only]														GT	794

Glasgow (1961)

		Totals
G. morsitans	Males	No record
	Female	No record

			Wing fray 1	2	3	4	5 or 6	6	Totals
G. swynnertoni	Males	Wild captures							1709
		Lab emergees							1697
	Female	Wild capture	623	302	255	424	117		1721
		Lab emergee	1711						1711

Glasgow & Bursell (1961)

	Year														Totals
G. swynnertoni	**1945**	0	0	0	0	0	0	0	0	15	221	108	89		433
	**1946**	98	102	52	301	201	178	247	89	58	98	68	50		1542
	**1947**	50	50	50	50	50	0	37	51	67	50	50	50		555
	**1948**	22	35	30	32	51	39	31	48	88	24	25	41		466
	**1949**	45	45	45	45	45	45	45	45	45	45	45	45		540
	**1950**	45	45	45	45	45	45	45	45	45	45	45	45		540
	**1951**	45	45	45	45	45	45	45	45	45	45	45	45		540
	**1952**	45	45	45	45	45	45	45	45	45	45	45	45		540
	**1953**	45	45	45	45	45	30	30	30	30	30	30	30		435
	**1954**	30	30	30	30	30	30	30	30	30	30	30	30		360
	**1955**	30	30	30	30	30	30	30	30	30	30	30	30		360
	**1956**	30	30	30	30	30	30	30	30	30	30	30	30		360
	**1957**	30	30	30	30	30	30	30	30	30	30	30	30		360
	**1958**	30	30	30	30	30	30	30	30	30	30	30	30		360
														GT	7391

Dransfield et al (1989)

			Month Aug	Sep	Oct	Nov	Dec	Jan	Feb	Totals
G. pallidipes	Males	Wild capture	349	380	429	44		311	136	1513
		Lab emergee	22	39	45	8		19	49	133
	Female	Wild capture	1090	679	647	253		593	229	3262
		Lab emergee	33	51	40	14		28	38	166

		Wing fray 1	2	3	4 to 6	Totals
Males	Wild capture	2266	4534	2664	1203	10 667
Female	Wild capture	2936	8013	5179	3177	19 305

		Ovarian category 0a	0b	1 to 3	4 to 7	Totals
Female		1132	1770	8947	7325	19 174

Text A. Limitations of our study

Sources of error The large proportion of the unexplained variation in regressions of individual fly sizes on meteorological variables, was probably due to various sources of uncertainty:

(i) ***Measurement error: differences between measurements obtained by various dissectors using a variety of microscopes***

Regression analysis demonstrated significant differences between egg length measurements made by five dissectors using eight different microscopes . Flies were distributed at random for processing by the various dissectors. Egg lengths were adjusted for wing length (*wlm*), the mean temperature (*Tbar91*) and NDVI (*ndvi91*) over the 9 days prior to the fly's capture date and the ovarian category of the adult female producing the egg.

Table P. Differences between mean lengths of samples of eggs of *G. pallidipes* as measured by five different dissectors (numbers 5, 6, 7, 9, 10) using nine different microscopes (numbers 1, 2, 9, 30, 31, 41, 51, 61, 71).

```
. regress ulm wlm tbar91 ndvi91 i.c i.di i.m if mdpall2<90 & di>4 & di<11 & di~=8 &
m~=40 & m~=50 & m~=60 & m~=70 & g==2 & s==2 & u==1 & c>0 & c<8 & ulm>130 &  ulm<200
& wlm>650 & wlm<800

      Source |       SS         df      MS    Number of obs = 53,279
-------------+----------------------------- F(21, 53257)  =  788.7
       Model |  657252.4        21   31297.7 Prob > F      =  0.000
    Residual |   2113499   53,257      39.7 R-squared     =  0.237
-------------+----------------------------- Adj R-squared =  0.237
       Total | 2770751.4   53,278      52.0 Root MSE      =  6.300
-------------------------------------------------------------------
         ulm |   Coef.  Std. Err.      t    P>|t|  [95% Conf. Int.]
-------------+-----------------------------------------------------
         wlm |   0.039    0.0012    31.56   0.000    0.037   0.042
      tbar91 |  -0.762    0.0100   -76.38   0.000   -0.782  -0.742
      ndvi91 |  13.253    0.189     70.11   0.000   12.883  13.624
           c |
          2  |   1.786    0.0999    17.88   0.000    1.591   1.982
          3  |   2.834    0.1022    27.72   0.000    2.634   3.034
          4  |   2.522    0.0952    26.48   0.000    2.335   2.708
          5  |   3.1842   0.1003    31.76   0.000    2.988   3.381
          6  |   2.9392   0.1065    27.60   0.000    2.730   3.148
          7  |   2.914    0.1279    22.79   0.000    2.664   3.165
          di |
          6  |  -6.542    0.1694   -38.61   0.000   -6.874   -6.210
          7  |  -2.096    0.1482   -14.14   0.000   -2.386   -1.805
          9  |  -4.514    0.1598   -28.24   0.000   -4.827   -4.200
         10  |  -2.463    0.1277   -19.28   0.000   -2.713   -2.213
           m |
          2  |   1.655    0.1261    13.12   0.000    1.408    1.902
          9  |   1.774    0.1658    10.70   0.000    1.449    2.099
         30  |  -0.405    0.1524    -2.66   0.008   -0.704   -0.106
         31  |   0.688    0.1388     4.96   0.000    0.416    0.961
         41  |   3.529    0.1643    21.48   0.000    3.207    3.851
         51  |  -0.360    0.1645    -2.19   0.029   -0.683   -0.038
         61  |   2.249    0.1813    12.41   0.000    1.894    2.604
         71  |  -1.031    0.1670    -6.17   0.000   -1.358   -0.704
       _cons | 155.4      1.0274   151.3    0.000  153.4    157.4
-------------------------------------------------------------------
```

Codes: *ulm* - Egg length (mm×100); *wlm* - Wing length (mm×100); *tbar91* - Mean temperature over 9 days prior to capture; *ndvi91* – Mean NDVI over 9 days prior to capture; *c* - Ovarian category; *di* - Dissector; *m* - Microscope; *mdpall2* - Capture method (excluding only laboratory flies.

(ii) Measurement error: problems due to remarkably small absolute variation in fly size

The difference between maximum and minimum monthly mean length values was only c. 0.5 mm and c. 0.2 mm for wing and egg lengths, respectively. This is illustrated in **Fig N**, mean lengths of wings and eggs of *G. pallidipes* are plotted on a scale that emphasises the small relative change in these lengths with season.

Microscope measurements, made even at the highest magnification, only allowed lengths to be estimated to the nearest 0.02 mm, i.e., about 10% of the difference between maximum and minimum egg length, even without variations between dissectors and microscopes.

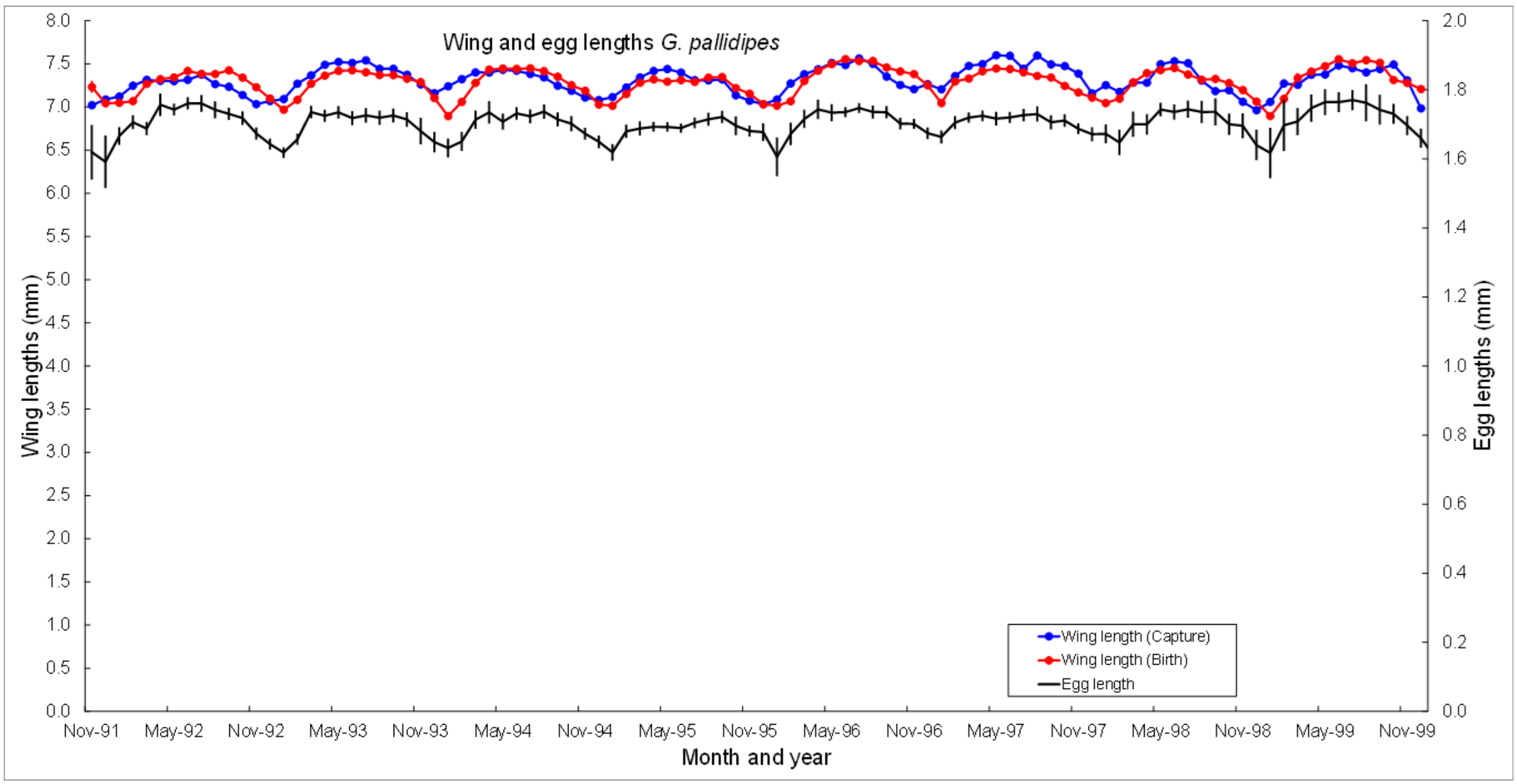

Fig N. Monthly variation in mean wing and egg lengths among female *G. pallidipes* sampled in the Zambezi Valley of Zimbabwe between November 1991 and December 1999.

This is a copy of **Fig I**, but with the scale on the Y-axis chosen to emphasise the small degree of variation in egg and wing lengths.

(iii) ***Uncertainty in the rates of development used in generating life histories***

We assumed no uncertainty in the rates of development used in generating life histories and assumed no effect of fly size and fly location on these rates. The models, applied to the Rekomitjie situation, were model fits to data, sometimes from laboratory flies. Moreover, in the absence of measured rates for *G. pallidipes* we used rates derived for *G. m. morsitans*. While temperature, the driver of the rates, is accurately measured by a weather station, the fly

will almost always be experiencing a different temperature (Vale, 1971; Torr & Hargrove, 1999). Violations of these assumptions, along with age estimation errors, may well lead to errors of a few to several days, in the timing of egg and wing developmental periods. However, wetness, greenness and temperature change slowly on a longer timescale. This may explain why we were so much more successful with modelling of weekly and monthly pooled data.

Whereas averaging the attributes of many flies over weeks or months provided useful predictive models, the disadvantage is that maternal attributes, such as size and ovarian age, can no longer be linked to individual daughters, thus inhibiting the understanding of maternal effects. Thus, while it seems certain that maternal size and age affect offspring size, and we see such effects from regressions carried out on individual flies, we cannot test these ideas on pooled samples.

(iv) Difficulties in estimating chronological ages

Carey et al (2012) point out that technologies for estimating the age of individual insects are generally accurate for young flies, less so for mid-aged individuals and that none can differentiate ages within the oldest age groups. Tsetse provide something of an exception in this regard: in this study we have assigned ages to flies up to ca. 35 days old – by which age we may expect that >50% of female tsetse will have died, even in favorable field conditions (Hargrove et al., 2011). We were, however, unable to assign an age to 20-30% of the dissected flies, because they had ovulated more than three times. Since wing and egg lengths varied little for flies where c >2 (Fig 7, main text), minimal bias should result from the loss of the older flies. Nonetheless, the inability to estimate accurately the chronological ages of older flies is undeniably a shortcoming of the ovarian dissection approach.

It has been suggested that mid-infrared spectroscopy (MIRS) and machine learning could be used for the accurate determination of chronological age in tsetse (Pazmiño-Betancourth et al., 2024). It is suggested further that the method could thus replace the use of ovarian dissection, which is viewed as labor-intensive, slow and inaccurate. Whereas, however, it is claimed that artificial intelligence could be used to discern the sex of a tsetse fly with 96% accuracy – natural intelligence and a cursory glance at the tip of the ventral abdomen would make the gender distinction with 100% accuracy. Moreover, the claimed ability of the technique to be able to separate female flies of ages 3 days and 5 weeks with 87% accuracy is

scarcely impressive. A trained dissector would, again, make the same distinction with close to 100% accuracy.

More importantly, the authors are missing the opportunity afforded by their new approach, which would come from using it in concert with, rather than replacing, ovarian dissection. Thus, whereas ovarian dissection cannot be used, by itself, to determine the number of times (c) a fly has ovulated if $c > 3$ it can identify this number modulo 4. For example, we do know that a fly where $c = 4+4n$ has ovulated exactly 4, 8, 12, 16 etc. times. At the start of these ovulation cycles a female, living at a mean temperature of 25°C, would be order 34, 71, 107, 143 days old, respectively. The MIRS method cannot yet match the accuracy of ovarian dissection in gauging the age of female flies that have ovulated <4 times. If, however, it could accurately separate any two females, differing in age by order 36 days, it could be used – together with ovarian dissection – to provide highly accurate estimates of the chronological ages, and indeed life histories, of female tsetse of all ages. One would then be able to produce the full life histories for all these flies – which would be a remarkable analytical breakthrough. We caution, however, that the same breakthrough was promised with the combined use of ovarian dissection and pteridine analysis (Lehane & Mail, 1985; Lehane & Hargrove, 1988) but the method has never been developed. The infra-red study must be repeated, using field flies of known chronological age, so that its true usefulness can be assessed.

References

Carey, J.R., Papadopoulos, N.T., Papanastasiou, S., Diamantidis, A., Nakas, C.T. (2012) Estimating changes in mean population age using the death distributions of live-captured medflies. Ecological Entomology 37, 359-369.

Hargrove, J. W. Ouifki, R., Ameh, J. E. (2011) A general model for mortality in adult tsetse (Glossina spp). Medical and Veterinary Entomology, 25, 385-394.

Lehane, M. J. & Hargrove, J. W. (1988) Field experiments on a new method for determining age in tsetse flies (Diptera: Glossinidae). Ecological Entomology 13, 319-322.

Lehane, M.J. & Mail, T.S. (1985) Determining the age of adult male and female Glossina morsitans morsitans using a new technique. Ecological Entomology, 10, 219-224.

Pazmiño-Betancourth, M., Casas Gómez-Uribarri, I., Mondragon-Shem, K., Babayan, S.A., Baldini, F., Rafuse Haines, L. (2024) Advancing age grading techniques for Glossina morsitans morsitans, vectors of African trypanosomiasis, through mid-infrared spectroscopy and machine learning. Biology Methods and Protocols, 9(1), p.bpae058.

Torr, S.J., Hargrove, J.W. (1999) Behaviour of tsetse (Diptera: Glossinidae) during the hot season in Zimbabwe: the interaction of micro-climate and reproductive status. Bulletin of Entomological Research, 89, 365-380.
